# BAI1 localizes AMPA receptors at the cochlear afferent post-synaptic density and is essential for hearing

**DOI:** 10.1016/j.celrep.2024.114025

**Published:** 2024-04-01

**Authors:** Adam J. Carlton, Jing-Yi Jeng, Fiorella C. Grandi, Francesca De Faveri, Ana E. Amariutei, Lara De Tomasi, Andrew O’Connor, Stuart L. Johnson, David N. Furness, Steve D.M. Brown, Federico Ceriani, Michael R. Bowl, Mirna Mustapha, Walter Marcotti

**Affiliations:** 1 School of Biosciences, University of Sheffield, Sheffield S10 2TN, UK; 2 Sorbonne Université, INSERM, Institute de Myologie, Centre de Recherche en Myologie, 75013 Paris, France; 3 Neuroscience Institute, University of Sheffield, Sheffield S10 2TN, UK; 4 School of Life Sciences, Keele University, Keele ST5 5BG, UK; 5 Mammalian Genetics Unit, MRC Harwell Institute, Harwell Campus, Oxfordshire OX11 0RD, UK

## Abstract

Type I spiral ganglion neurons (SGNs) convey sound information to the central auditory pathway by forming synapses with inner hair cells (IHCs) in the mammalian cochlea. The molecular mechanisms regulating the formation of the post-synaptic density (PSD) in the SGN afferent terminals are still unclear. Here, we demonstrate that brain-specific angiogenesis inhibitor 1 (BAI1) is required for the clustering of AMPA receptors GluR2–4 (glutamate receptors 2–4) at the PSD. Adult *Bai1*-deficient mice have functional IHCs but fail to transmit information to the SGNs, leading to highly raised hearing thresholds. Despite the almost complete absence of AMPA receptor subunits, the SGN fibers innervating the IHCs do not degenerate. Furthermore, we show that AMPA receptors are still expressed in the cochlea of *Bai1*-deficient mice, highlighting a role for BAI1 in trafficking or anchoring GluR2–4 to the PSDs. These findings identify molecular and functional mechanisms required for sound encoding at cochlear ribbon synapses.

## Graphical abstract

**Graphic abstract:**
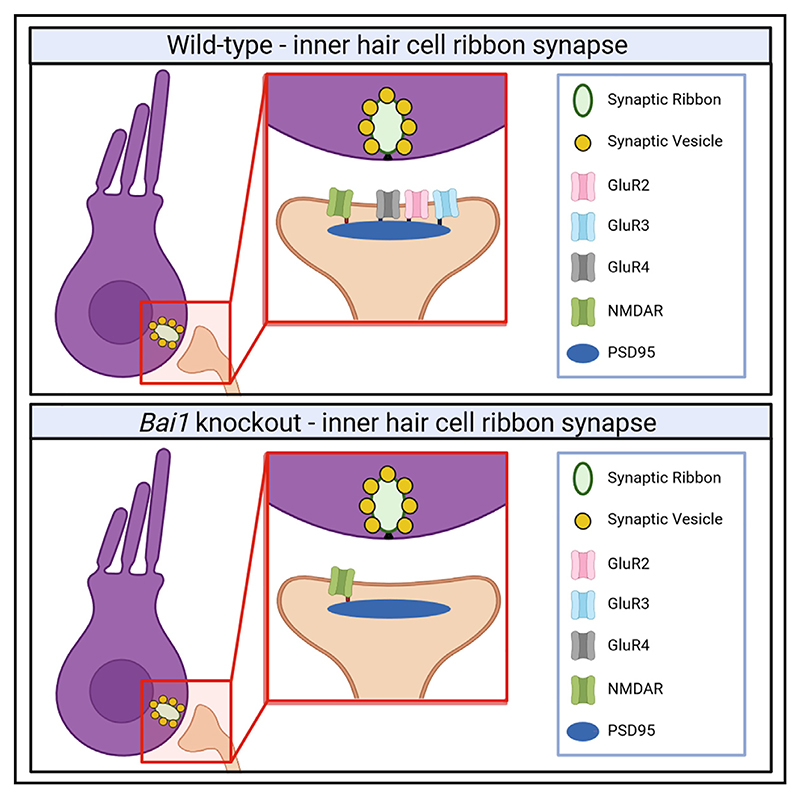

## Introduction

In mammals, a precise representation of the auditory landscape requires the processing of acoustic stimuli with unparalleled temporal precision (in the range of μs) over a wide range of sound intensity and frequency.^1,2^ While sound frequency is mainly encoded by the tonotopic organization of the sensory hair cells along the length of the cochlea,^3^ the intensity and timing of the sound waveform are largely defined by the firing characteristics of the auditory afferent fibers.^4^ Type I spiral ganglion neurons (SGNs) represent the majority of the afferent fibers that innervate the cochlea (~95%),^5^ the role of which is to relay sound information toward the brain. Each type I afferent fiber makes a single bouton-like synapse with an inner hair cell (IHC), which are the primary auditory receptors of the mammalian cochlea. In mice, each IHC is normally contacted by up to 20 SGN afferent boutons that are paired with a pre-synaptic ribbon,^6,7^ the role of which is to tether vesicles to facilitate high rates of sustained synaptic transmission.^8,9^ The physiological characteristics of SGN afferent fibers are very heterogeneous, showing a wide range of thresholds and spontaneous firing rates,^10–12^ which allow them to convey the wide dynamic range of sound intensity encoded by the IHCs.^2^

Recent studies using single-cell RNA sequencing (scRNA-seq) on the mammalian cochlea have identified several genes that are expressed in SGNs,^13–15^ including the brain-specific angiogenesis inhibitor *Bai1*. The BAI family, a subclass of adhesion G-protein-coupled receptors, consists of three members.^16^ BAI1, which is encoded by the *ADGRB1* (adhesion G-protein-coupled receptor B1) gene,^17^ was initially identified as a target of the tumor suppressor p53.^18,19^ BAI1 has also been shown to play crucial roles in diverse cellular processes such as suppressing angiogenesis,^20^ promoting myogenesis,^21^ and the internalization of apoptotic cells.^22^ Knockdown of BAI1 has been shown to affect synaptogenesis in hippocampal and cortical neurons.^23^ Furthermore, mice lacking *Bai1* have been shown to display reduced expression of PSD-95 and a thinning of the post-synaptic density (PSD) in hippocampal neurons^24^ leading to deficits in spatial learning and memory^24^ and brain development and an increased susceptibility to seizures.^25^ Moreover, a rare *BAI1* variant has been identified in patients affected with autism spectrum disorders,^26^ and *Bai1* has been suggested to be linked to hearing loss in mice.^27^ Despite the important role of BAI1 in the CNS, the mechanism(s) by which it mediates afferent synaptogenesis is still poorly understood.

In this study, we investigated the role of *Bai1* (*Adgrb1*) in the auditory system using *Bai1*-deficient (*Bai1*^*tm2b*^) mice generated by the International Mouse Phenotyping Consortium. We found that BAI1 is expressed in cochlear afferent SGNs. Using a combination of functional, morphological, and molecular approaches, we found that BAI1 is required for the correct localization of AMPA receptors (GluR2–4 [glutamate receptors 2–4]) to the PSD. Transcriptomic analysis also reveals that the absence of functional *Bai1* leads to many gene expression changes that are also found in *VGlut3* knockout mice and highlights a role for BAI1 in trafficking or anchoring GluR2–4 to the PSDs.

## Results

**Figure 1:**
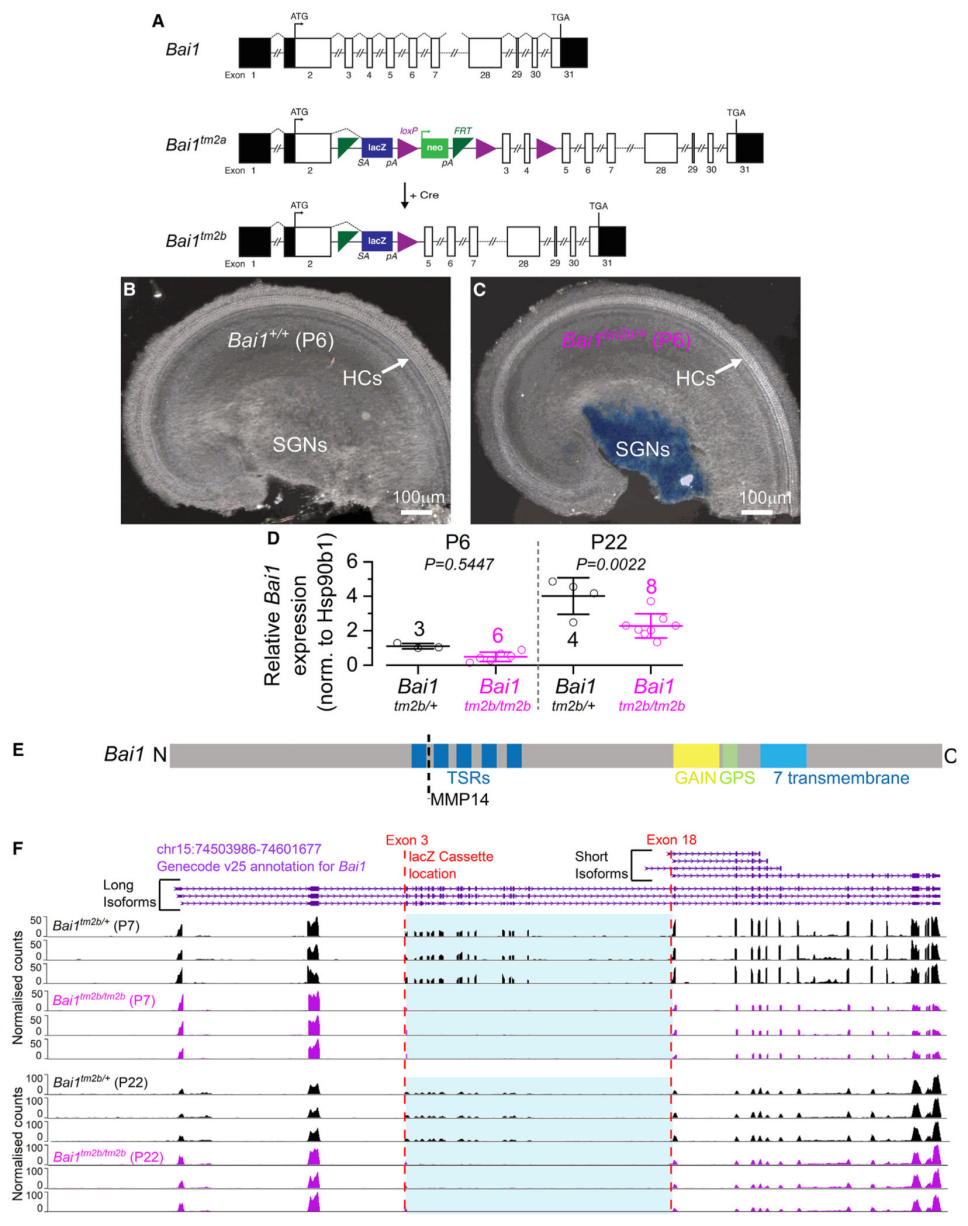
Generation of *Adgrb1*-deficient mice (*Bai1*^*tm2b/tm2b*^) and *Adgrb1* expression in the mouse cochlea (A) Schematic representation of the genomic structure of the mouse adhesion G-protein-coupled receptor (GPCR) B1 (*Adgrb1*) gene (ENSMUSG00000034730; MGI: 1933736). The gene comprises 31 exons spanning ~73 Kb of genomic DNA on chromosome 15. Adgrb1 is a 1,582-amino-acid 7-transmembrane protein with an extended extracellular region. The ATG (translation start) and the TGA (stop) sites are in exons 2 and 31, respectively, and the untranslated regions are shown in black. The International Mouse Phenotyping Consortium (IMPC) uses different targeting strategies to produce knockout alleles, which rely on the identification of critical exons common to all transcript variants that, when deleted, disrupt gene function.^28^ For the *Adgrb1* gene, a promoter-driven targeting cassette was used to generate a knockout-first allele (tm2a) in C57BL/6N embryonic stem cells. Insertion of the lacZ trapping cassette and a floxed promoter-driven *neo* cassette inserted into intron 2 of the gene is expected to disrupt gene function. Cre-mediated deletion of the selection cassette and floxed exons 3 and 4 of the tm1a allele generates a lacZ-tagged allele (tm2b), which was used for the present study. FRT, flippase recognition target; *neo*, neomycin resistance gene; pA, polyadenylation site; SA, splice acceptor. (B and C) X-gal staining of the cochlear apical-coil region from P6 control and heterozygous littermate mice showing strong *Adgrb1* expression (blue) in the cell body of spiral ganglion neurons (SGNs). Images are examples from eight control and eleven *Bai1*^*tm2b/+*^ mice. Note that we cannot exclude the possible presence of X-gal staining in the satellite glial cells. However, inner and outer hair cells (IHCs and OHCs, respectively) were not stained with X-gal. (D) qPCR showing the expression of *Bai1* in the apical coil of the *Bai1*^*tm2b/+*^ and littermate *Bai1*^*tm2b/tm2b*^ mouse cochlea. Number of replicas is shown above the data, and each replica contains cochleae from 3–4 mice (mean ± SD). (E) Diagram of protein domains of mouse BAI1 protein. The lacZ cassette removes the thrombospondin type-1 repeats (TSRs) but leaves intact the GPCR-autoproteolysis inducing (GAIN), GPCR proteolytic site (GPS), and transmembrane domains. (F) Normalized reads from P7 and P22 control (*Bai1*^*tm2b/+*^) and knockout (*Bai1*^*tm2b/tm2b*^) mice. Three out of seven representative animals from 2 batches were chosen for each genotype. Top trace shows the Genecode annotations for the *Bai1* (*Adgrb1*) isoforms. The lacZ cassette removes reads from exons 3–18 but leaves the short isoform intact. See also Figures S1 and S2.

*Bai1*-deficient (*Bai1*^*tm2b*^) mice of both sexes were produced through Cre-mediated conversion of the “knockout-first” tm2a allele, which was achieved by treating *in*-*vitro*-fertilization-derived embryos with a cell-permeable Cre enzyme (Figure 1). In the converted tm2b allele (*Bai1*^*tm2b*^), exons 3 and 4 (ENSMUSE00001058436 and ENSMUSE00000963718) of the *Adgrb1* gene (ENSMUSG00000034730; MGI: 1933736), located on chromosome 15, are deleted, leaving a lacZ reporter cassette containing a splice acceptor that subsumes normal splicing (Figure 1A). X-gal staining of the cochlea from postnatal day 6 and 7 (P6–P7) mice showed that *LacZ* is expressed in the cell body of the SGNs (Figures 1B and 1C). scRNA-seq data have also shown that *Bai1* is expressed in all subtypes of SGNs from P25–P27 mice with no change along the cochlear tonotopic axis (Figure S1).^14^ qPCR analysis from P6 and P22 cochlear tissue revealed that *Bai1* is significantly downregulated in *Bai1*^*tm2b/tm2b*^ mice compared to controls (*Bai1*^*tm2b/+*^) only at the older age tested (Figure 1D). *Bai1* has at least two isoforms: a long form, which contains the extracellular thrombospondin repeats (TSRs), and a short form, which contains the intracellular domains only^16^ (Figures 1E and 1F). To determine which, if any, of the *Bai1* isoforms are affected by the tm2b allele, we performed RNA-seq of bulk cochlear tissues and visualized the mapped reads on the *Bai1* gene. As expected, in the heterozygous animals, we could observe reads mapping to both the long isoform as well as shared regions of the short isoform. However, splicing analysis of the RNA-seq data in homozygous *Bai1*^*tm2b*^ mice at both P7 and P22 showed a loss of reads mapping between exon 3, where the lacZ cassette inserted, and exon 18, where the short isoform(s) begins (Figure 1F), leaving an intact short isoform with no observed differences in expression levels (Figure S2). Therefore, we concluded that the *Bai1*^*tm2b*^ mice represent a knockout model for the long isoform of *Bai1*.

### *Bai1^tm2b/tm2b^* mice exhibit early-onset hearing loss

**Figure 2:**
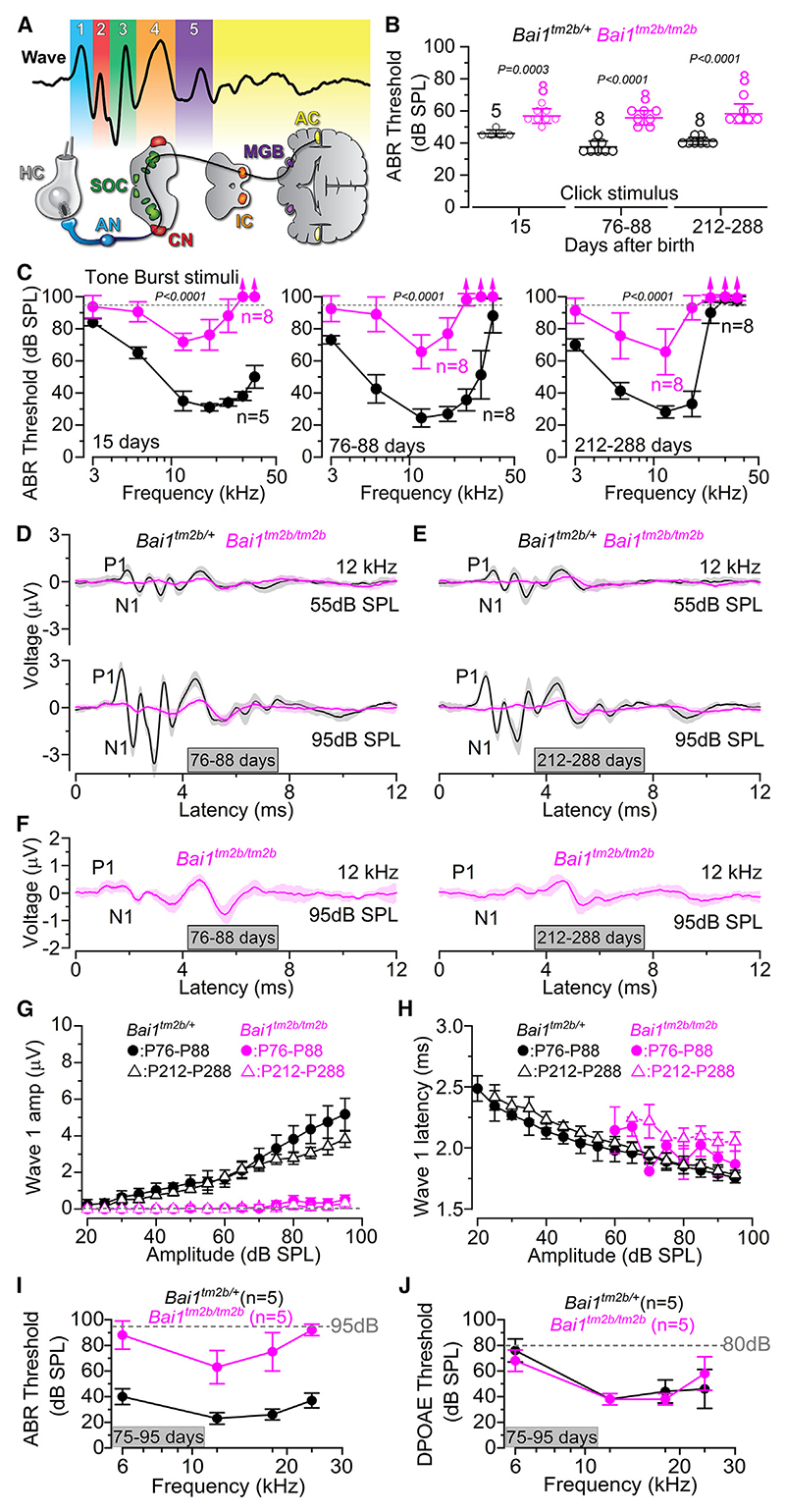
Auditory brainstem response (ABR) thresholds, but not distortion product otoacoustic emissions (DPOAEs), are elevated in *Bai1* mice (A) Schematic representation showing the ABR waveform and the corresponding neuronal component along the ascending auditory pathway. Wave 1 represents the cochlear output and is generated by the auditory afferent fibers (AFs), wave 2 by the cochlear nucleus (CN), wave 3 by the superior olivary complex (SOC), and wave 4 by the lateral lemniscus and inferior colliculus (IC). (B) Average ABR thresholds elicited by click stimuli applied to control (*Bai1*^*tm2b/+*^) and knockout littermate mice (*Bai1*^*tm2b/tm2b*^) at three age ranges: P15, P76–P88, and P212–P288. Data are plotted as mean values ± SD. Number of mice (biological replica) used is shown above the averages, and single data points are plotted as open circles. Each mouse was only tested once (technical replica). Both biological and technical replicates apply to (B)–(H). Statistical values: Tukey’s post-test, one-way ANOVA. (C) ABR thresholds for frequency-specific pure-tone stimulations ranging from 3 to 36 kHz recorded from *Bai1*^*tm2b/+*^ and *Bai1*^*tm2b/tm2b*^ littermate mice (age as in B). Data are plotted as mean values ± SD. Numbers of mice tested are shown next to the traces. Statistical test: two-way ANOVA. (D and E) Average ABR waveform responses at 12 kHz at increasing stimulus intensity (dB sound pressure level: dB SPL) at P76–P88 (D) and P212–P288 (E) obtained from the same mice listed above. Continuous lines represent the average values and the shaded areas represent the SD. P1 and N1 indicate the positive and negative peaks of wave 1, respectively. (F) Expanded view of the average ABR waveform responses at 12 kHz and the highest sound intensity (95 dB) in *Bai1*^*tm2b/tm2b*^ (from C) at P76–P88 (left) and P212–P288 (right). (G and H) Average amplitude (G: from P1 to N1: see D–G) and latency of wave 1 (H: time between the onset of the stimulus and P1) as a function to the actual dB SPL sound intensity recorded from adult *Bai1*^*tm2b/+*^ and *Bai1*^*tm2b/tm2b*^ mice at the two age ranges (P76–P88 and P212–P288). Data are plotted as mean values ± SD. Note that most of the wave 1 amplitude data in *Bai1*^*tm2b/tm2b*^ mice (G) are near zero, as indicated by the dashed gray line. (I and J) ABR (I) and DPOAE (J) thresholds measured from the same *Bai1*^*tm2b/+*^and *Bai1*^*tm2b/tm2b*^ adult mice at P75–. Data are plotted as mean values ± SD. P95. The frequency range tested: 6, 12, 18, and 24 kHz. The dashed line represents the upper threshold limit used for ABRs (95 dB) and DPOAEs (80 dB). Note the normal DPOAE thresholds in *Bai1*^*tm2b/tm2b*^ mice despite the highly elevated ABR thresholds. Five mice per genotype were used for both ABRs and DPOAEs (I and J). See also Figure S3.

Auditory brainstem responses (ABRs) were used to test the hearing sensitivity of *Bai1*^*tm2b*^ mice (Figure 2A). ABR thresholds were defined as the lowest sound level where any recognizable wave was visible. Control mice (*Bai1*^*tm2b/+*^) showed normal thresholds to click stimuli at all ages tested, as previously shown in wild-type mice,^29^ with a characteristic improvement in sound pressure threshold between early post-hearing ages (P15) and older mice (P76–P88; p = 0.0002, Tukey’s post-test from one-way ANOVA, Figure 2B). The thresholds remained relatively stable up to at least 212–288 days of age (p = 0.0517, Tukey’s post-test). Thresholds for clicks recorded from the long-isoform *Bai1* knockout mice (*Bai1*^*tm2b/tm2b*^) did not change significantly between P15 and P212–P288 (p = 0.2846, one-way ANOVA), although they were raised at all three ages tested (p < 0.0001, two-way ANOVA) compared to control mice (Figure 2B). Pure-tone-evoked ABRs were also found to be significantly elevated in all age groups tested in *Bai1*^*tm2b/tm2b*^ compared to littermate controls (p < 0.0001 for all ages: two-way ANOVA, Figure 2C). These results also show that *Bai1*^*tm2b/tm2b*^ mice do not exhibit any progressive worsening of hearing with age. The hearing loss occurring at high frequencies (>12 kHz) is due to the C57BL/6 mouse strain harboring a hypomorphic allele in *Cadherin 23* (*Cdh23*^*ahl*^), which is also present in control mice.^30^

To better assess the sound-induced output of the cochlea, we analyzed ABR wave 1 that is generated by the summed response to the sound of all afferent nerve fibers innervating the IHCs.^31,32^ ABR wave 1 was analyzed for 12 kHz responses (Figures 2D–2F), as this closely matches the cochlear region used for the following *ex vivo* experiments (9–12 kHz). We also found that wave 1 amplitude in *Bai1*^*tm2b/tm2b*^ was close to zero across the wide range of sound intensities tested and at both ages (p < 0.0001 compared to control mice, Tukey’s post-test, two-way ANOVA, Figures 2D, 2E, and S3A). Despite the almost complete loss of cochlear output, the subsequent waves of the ABR recordings were more easily detected due to auditory central gain.^33,34^ The latency of the residual wave 1 in *Bai1*^*tm2b/tm2b*^ was, however, not significantly different between the two genotypes at both ages (p > 0.9999, two-way ANOVA, Figure 2H). We then recorded distortion product otoacoustic emissions (DPOAEs), which are a product of cochlear amplification caused by sound-induced outer hair cell (OHC) electromotility and therefore provide a specific readout of OHC function. For this experiment, the same adult mice were tested for both ABRs and DPOAEs. We found that despite the largely elevated ABR thresholds, DPOAE thresholds over the same age range were indistinguishable from those recorded from littermate controls (p = 0.8659, two-way ANOVA, Figures 2J and S3B), indicating that OHCs are fully functional. These results suggested that the loss of the long isoform of *Bai1* (*Adgrb1*) is likely to cause auditory neuropathy by affecting the activity of either the IHCs and/or that of the auditory afferent fibers.

### *Bai1* is not required for hair cell function

**Figure 3:**
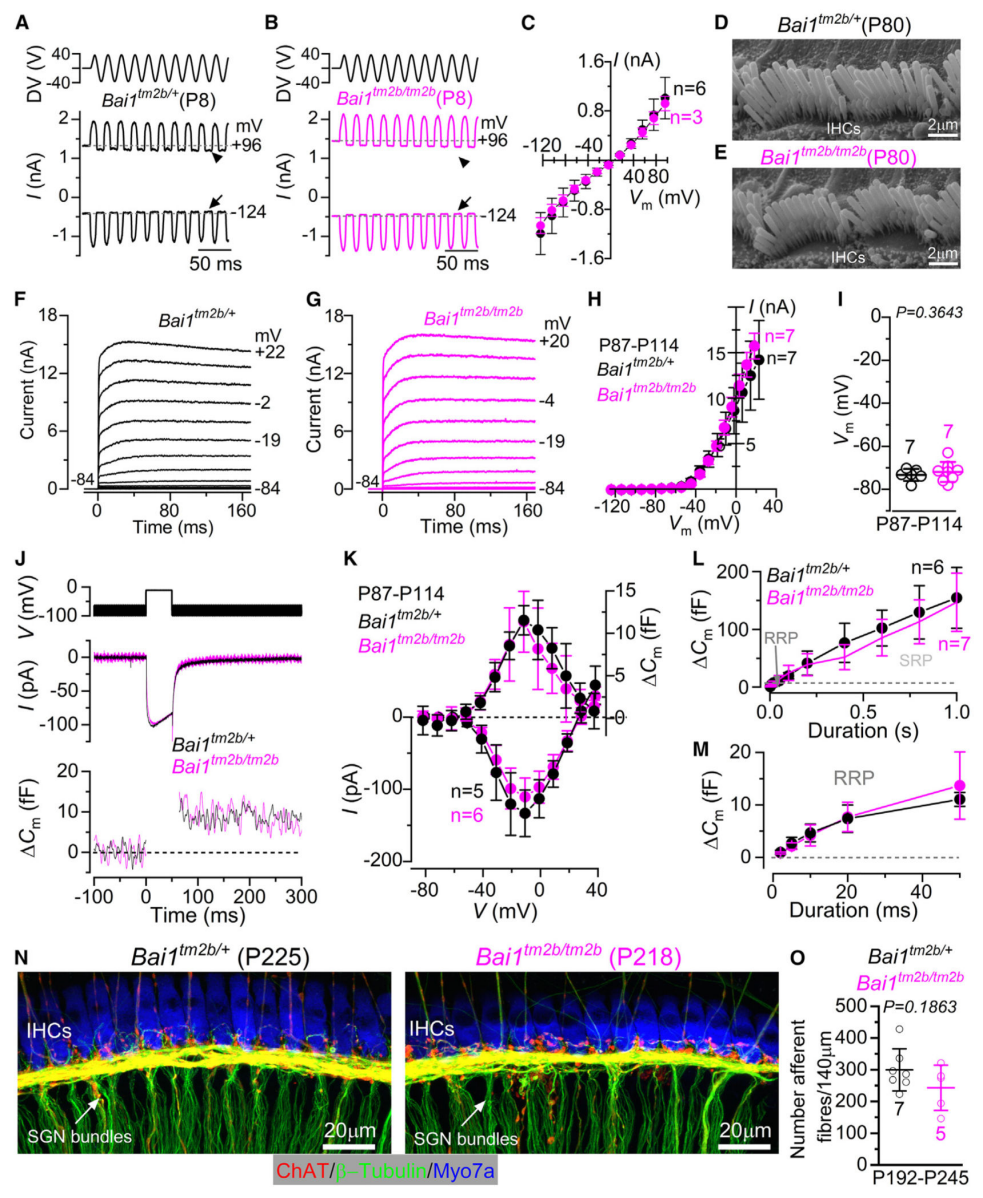
IHC function is normal in *Bai1* mice (A and B) Saturating mechanoelectrical transducer (MET) current in apical IHCs from *Bai1*^*tm2b/+*^ (A) and *Bai1*^*tm2b/tm2b*^ (B) P8 mice in response to 50 Hz sinusoidal force stimuli to the hair bundles at two membrane potentials. DV: driver voltage applied to the fluid. Arrows and arrowheads: closure of the MET channel at –124 and +96 mV, respectively. (C) Average peak-to-peak MET current-voltage curves recorded by displacing the hair bundles of IHCs from both genotypes while stepping their membrane potential from –124 to +96 mV in 20-mV increments (p = 0.8842, two-way ANOVA). Data are plotted as mean values ± SD. (D and E) Scanning electron micrographs (SEMs) showing the typical hair bundle staircase structure with 3 rows of stereocilia in IHCs from *Bai1*^*tm2b/+*^ (D) and *Bai1*^*tm2b/tm2b*^ (E) P80 mice (examples from 3 mice per genotype). (F and G) Current responses from IHCs of *Bai1*^*tm2b/+*^ and *Bai1*^*tm2b/tm2b*^ adult mice elicited by applying depolarizing voltage steps (10 mV nominal increments) from –84 mV to the various test potentials shown next to some of the traces. (H) Steady-state current-voltage (*I-V*_m_) curves obtained from IHCs of both genotypes at P87–P114 (p = 0.3094, two-way ANOVA). Data are plotted as mean values ± SD. (I) Resting membrane potential (*V*_m_) measured from P87–P114 IHCs of both genotypes (statistical comparisons: t test). Data are plotted as mean values ± SD. (J and K) Calcium current (*I*_Ca_) and corresponding changes in membrane capacitance (*ΔC*_m_) recorded from IHCs of both genotypes (P87–P114) in response to 50 ms voltage steps (10 mV increments) from -81 mV. In (J), only maximal responses at –11 mV are shown. (K) shows average peak *I*_Ca_ (bottom) and *ΔC*_m_ (top) curves from both genotypes. Data are plotted as mean values ± SD. (L) Average *ΔC*_m_ from IHCs of both genotypes (P87–P114) in response to voltage steps from 2 ms to 1 s showing the RRP and SRP. Data are plotted as mean values ± SD. (M) RRP (expanded from L). Data are plotted as mean values ± SD. (N) Maximum intensity projections of confocal z stacks of the fibers innervating the IHCs from 7- to 8-month-old *Bai1*^*tm2b/+*^ and *Bai1*^*tm2b/tm2b*^ mice. The cochlea was immunolabeled using antibodies against β-tubulin (afferent and efferent fiber marker) and ChAT (efferent fiber marker). Myosin 7a (Myo7a) was used as the IHC marker. (O) Number of AFs from *Bai1*^*tm2b/+*^ and *Bai1*^*tm2b/tm2b*^ mice, which were those β-tubulin positive and ChAT negative (statistical comparisons: t test). Numbers above the mean data indicate the mice used for each genotype. Data are plotted as mean values ± SD. Data in (A)–(C): control, 6 IHCs from 2 mice; *Bai1*^*tm2b/tm2b*^, 3 IHCs from 1 mouse. Data in (F)–(I): control, 7 IHCs from 4 mice; *Bai1*^*tm2b/tm2b*^, 7 IHCs from 3 mice. Data in (J) and (K): control, 5 IHCs from 3 mice; *Bai1*^*tm2b/tm2b*^, 6 IHCs from 3 mice. Data in (L) and (M): control, 6 IHCs from 3 mice; *Bai1*^*tm2b/tm2b*^, 7 IHCs from 3 mice. Data in (N) and (O): control, 89 afferent bundles from 7 mice; *Bai1*^*tm2b/tm2b*^, 60 afferent bundles from 5 mice). See also Figures S4–S8.

Although our X-gal staining revealed that *Bai1* is only present in SGNs (Figure 1C), scRNA-seq gene expression profiling (gEAR: https://umgear.org/) has indicated its presence in both IHCs and OHCs. We focused our investigation on IHCs because the function of OHCs was normal in *Bai1*^*tm2b/tm2b*^ mice (Figure 2J). The mechanoelectrical transducer (MET) current from P8 apicalcoil IHCs was elicited by displacing their stereociliary bundles using a 50 Hz sinusoidal force stimulus from a piezo-driven fluid jet.^35^ IHCs from both control and *Bai1*^*tm2b/tm2b*^ mice showed a MET current with biophysical characteristics indistinguishable between the two genotypes (Figures 3A–3C and S4). This finding was also supported by the normal staircase morphology of the hair bundles in adult IHCs from both genotypes (Figures 3D, 3E, and S5).

In mature IHCs, the size of the basolateral membrane K^+^ currents and resting membrane potential were indistinguishable between *Bai1*^*tm2b/+*^ and *Bai1*^*tm2b/tm2b*^ littermate P87–P114 mice (Figures 3F–3I and S6). Pre-synaptic activity or exocytosis in adult IHCs was estimated by measuring the size of the Ca^2+^ current (*I*_Ca_) and the induced increase in cell membrane capacitance (*ΔC*_m_) following depolarizing voltage steps (Figures 3J and 3K). The sizes of *I*_Ca_ and *ΔC*_m_ were not significantly different between the two genotypes (p = 0.0658 and 0.4257, respectively, two-way ANOVA, Figure 3K). The rate of neurotransmitter release in adult IHCs was investigated by measuring *ΔC*_m_ in response to depolarizing voltage steps to –11 mV of varying duration between 2 ms and 1 s (interstep interval was at least 11 s) (Figures 3L and 3M). Under our experimental conditions (1.3 mM extracellular Ca^2+^ and body temperature), stimuli up to about 50 ms reveal the readily releasable pool (RRP) of the vesicle, while longer steps induce the release of vesicles from a secondarily releasable pool (SRP) that is located farther away from the Ca^2+^ channels.^36^ We found that both the RRP (Figure 3M) and the SRP (Figure 3L) recorded from the IHCs of *Bai1*^*tm2b/tm2b*^ mice were not significantly different from those obtained in control *Bai1*^*tm2b/+*^ mice (p = 0.5899 and 0.1757, respectively, two-way ANOVA). Overall, the above findings show that the long isoform of *Bai1* is not required for the development and function of IHCs.

### Afferent fibers and terminals are present in *Bai1^tm2b/tm2b^* mice

The almost complete absence of wave 1 in the ABR waveforms recorded from *Bai1*^*tm2b/tm2b*^ mice (Figures 2D–2G) could be explained by the loss of the SGNs and their afferent fibers and/or their synapses. Since the SGN marker β-tubulin also labels the efferent fibers, the afferent fibers were identified as β-tubulin positive but negative to an antibody targeting choline acetyltransferase (ChAT) that specifically labels the efferent system (Figure 3N). Using a 3D reconstruction of the SGN bundles, we found that the number of afferent fibers in the apical region of the cochlea from adult *Bai1*^*tm2b/tm2b*^ mice was not significantly different from that measured in littermate *Bai1*^*tm2b/+*^ mice at least up to 8 months of age (Figure 3O). In line with these findings, the SGN somata were also present in adult *Bai1*^*tm2b/tm2b*^ mice (Figure S7). The ChAT-positive efferent fibers and the synaptic vesicle protein 2 at the efferent endings were also present in both genotypes (Figure S8). These results indicate that the absence of long isoform of *Bai1* does not affect the survival of the SGNs.

**Figure 4:**
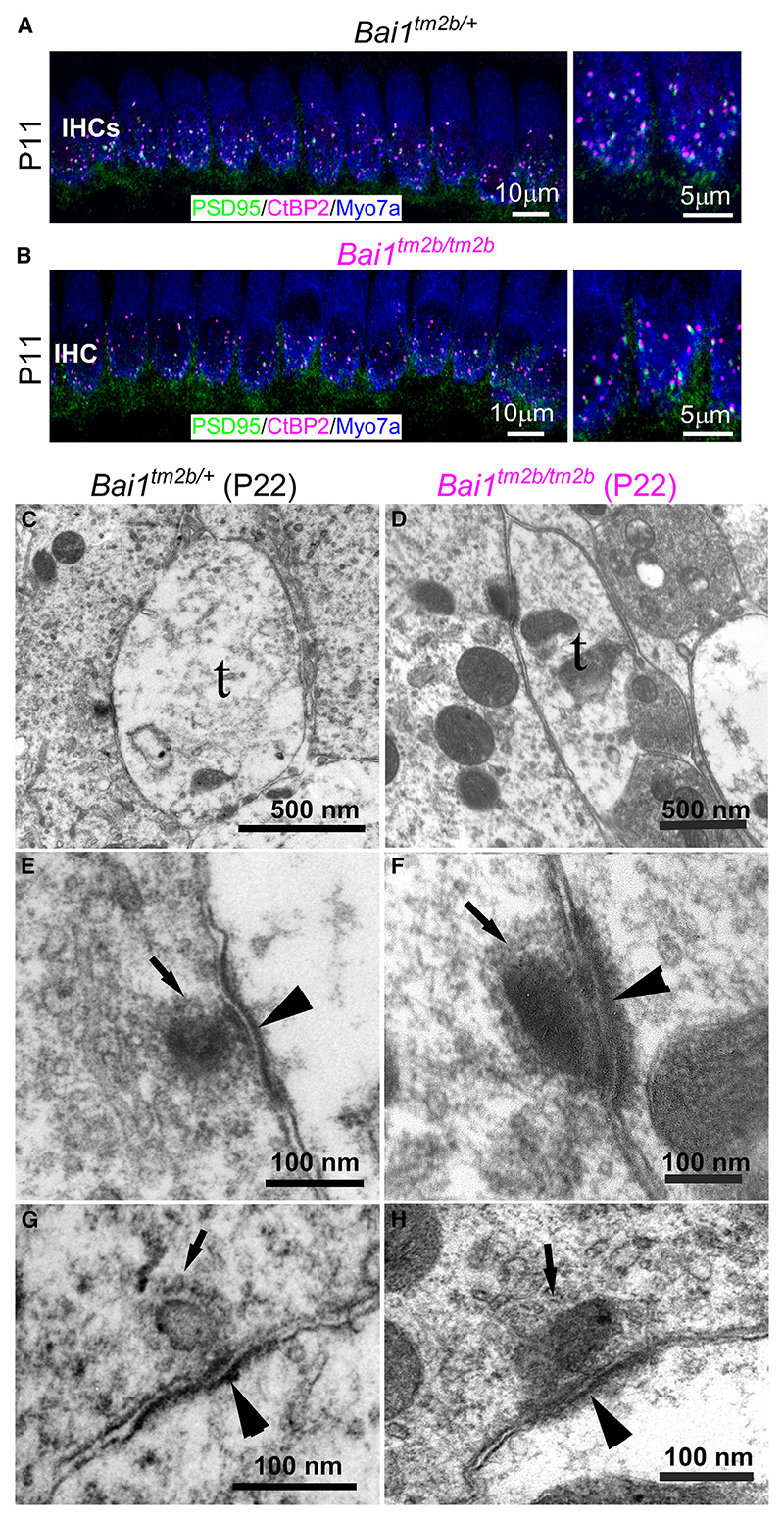
Post-synaptic densities at the afferent terminals are not affected in *Bai1*-deficient mice (A and B) Maximum intensity projections of confocal z stacks of IHCs taken from the apical cochlear region of *Bai1*^*tm2b/+*^ (A) and *Bai1*^*tm2b/tm2b*^ (B) mice using antibodies against the post-synaptic density protein PSD-95 and the ribbon marker CtBP2. Myo7a: IHC marker. Enlarged views (right) show the colocalization of PSD-95 and CtBP2. (C–H) Transmission electron microscopy of synaptic structures in the *Bai1*^*tm2b/+*^ and *Bai1*^*tm2b/tm2b*^ mice. Low-power image (C) shows the typical ribbon synaptic structure with the terminal (t) of the afferent nerve containing light cytoplasm compared with that of the IHC (left of image). Note the dark synaptic bar adjacent to the apposed membranes on the IHC side. (D) shows a similar view of the synaptic region for the *Bai1*^*tm2b/tm2b*^: terminal with synaptic bar in the IHC adjacent to the apposed synaptic membranes. The higher-magnification images (E–H) show two afferent terminals from *Bai1*^*tm2b/+*^ (E and G) and two from *Bai1*^*tm2b/tm2b*^ mice (F and H). The bar and synaptic cleft are visible in each image with post-synaptic density (arrowheads) and synaptic vesicles around the bar (arrows). Images in (A) and (B) are examples from 3 mice per genotype. Data in (C)–(H) were obtained from 10 post-synaptic bars from 3 mice (control) and 11 afferent bundles from 3 mice (*Bai1*^*tm2b/tm2b*^). See also Figure S9.

Mice lacking *Bai1* (full knockout) have been shown to have reduced expression of the PSD component PSD-95 at hippocampal synapses.^24^ Therefore, we investigated whether the localization of PSD-95 and SHANK-1, which is another key protein expressed in the PSD of glutamatergic synapses in the CNS and cochlea,^37,38^ was affected in *Bai1*^*tm2b/tm2b*^ mice. Immunofluorescence labeling also showed that PSD-95 was normally distributed at the IHC ribbon synapses, being juxtaposed to the pre-synaptic marker CtBP2 in both control and *Bai1*^*tm2b/tm2b*^ P11 mice (Figures 4A and 4B). The post-synaptic protein SHANK-1 was also expressed in the basal pole of the IHCs from both genotypes (Figure S9). transmission electron microscopy experiments on P22 mice (3 mice per genotype) indicated no obvious structural differences in the pre-synaptic ribbons and the PSD between *Bai1*^*tm2b/+*^ and *Bai1*^*tm2b/tm2b*^ mice (Figures 4C–4H). The length of the PSDs was similar between *Bai1*^*tm2b/+*^ (298 ± 83 nm, n = 10) and *Bai1*^*tm2b/tm2b*^ (319 ± 70 nm, n = 11, p = 0.5182, t test) mice. These results demonstrate that afferent fibers and PSD are preserved in adult *Bai1*^*tm2b/tm2b*^ mice.

### The SGN PSD of adult *Bai1^tm2b/tm2b^* mice is almost completely devoid of AMPA GluRs

**Figure 5:**
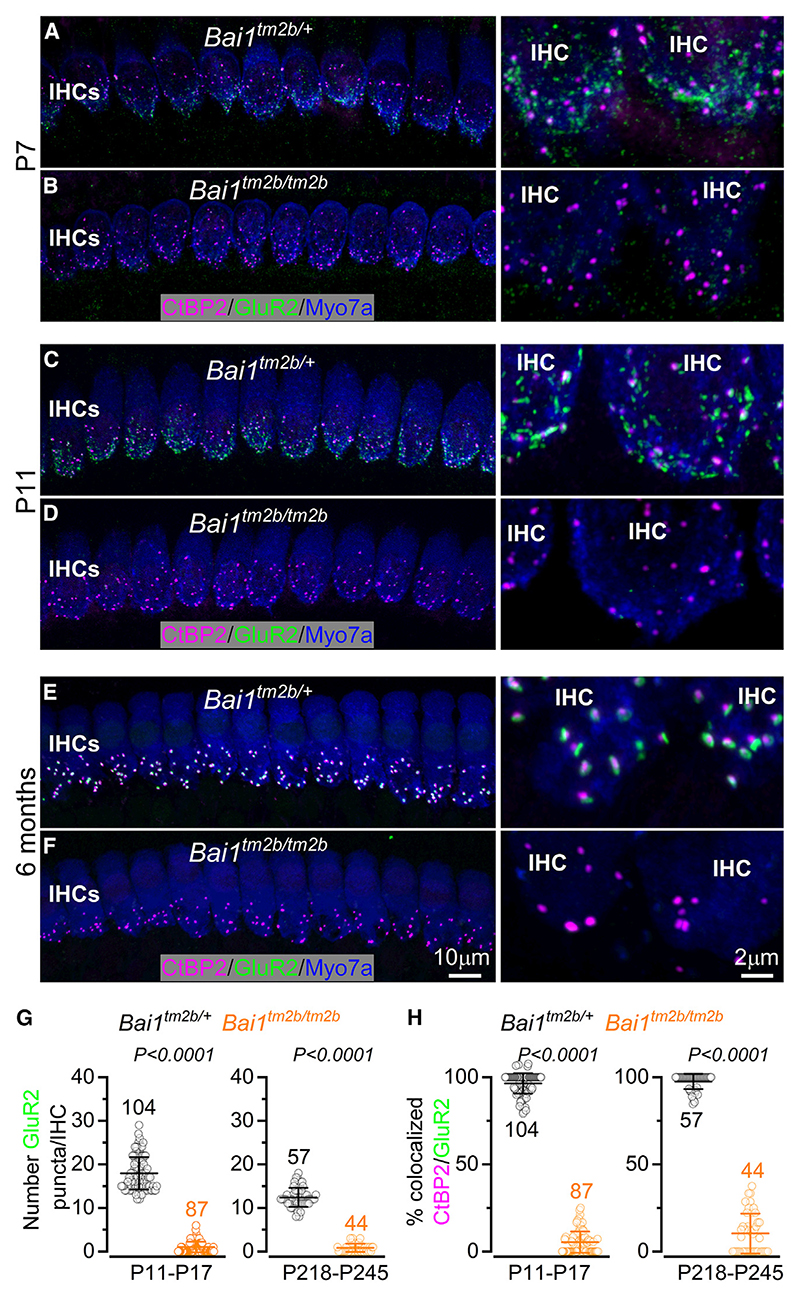
Expression of AMPA-type GluR2 receptors in the IHCs of *Bai1* mice (A–F) Maximum intensity projections of confocal z stacks of apical-coil IHCs of *Bai1*^*tm2b/+*^ and *Bai1*^*tm2b/tm2b*^ mice at pre-hearing (P7, A and B; P11, C and D) and adult ages (P218–P245, E and F) using antibodies against CtBP2 (ribbon synaptic marker) and GluR2 (post-synaptic marker). Myo7a: IHC marker. Right columns are enlarged views of the IHC synaptic region showing the level of colocalization between CtBP2 and GluR2 puncta. Scale bars shown in (F) also apply to (A)–(E). (G) Number of GluR2 puncta present at the IHC synaptic region at two age ranges in control and *Bai1*^*tm2b/tm2b*^ mice. Data are plotted as mean values ± SD. Note that P17 is not shown in images listed in (A)–(F). (H) Percentage of CtBP2 and GluR2 colocalization at P11–P17 and P218–P245 from both genotypes (number of mice as in G). Data are plotted as mean values ± SD and individual GluR2 counts (smaller open symbols). Numbers above the mean data indicate the IHCs used from P11–P17 and P218–P245: *Bai1*^*tm2b/+*^ (7 and 4 mice, respectively); *Bai1*^*tm2b/tm2b*^ (6 and 3 mice). Statistical values shown in (G) and (H) were obtained using a Mann-Whitney U test.

Cochlear IHCs transmit sound-induced information to the SGNs via the release of glutamate, which primarily activates AMPA-type glutamatergic receptors at the post-synaptic afferent terminals.^9,39^Therefore, we assessed whether the strongly reduced wave 1 in the ABR recordings (Figure 2) was due to defects in the number and/or localization of GluRs at the PSDs. In the adult cochlea, afferent neurons appear to express only three of the four AMPA-type pore-forming subunits GluR2–4.^40–42^ We found that, in contrast to control mice, SGNs from both pre- and post-hearing *Bai1*^*tm2b/tm2b*^ mice showed very few or no GluR2 puncta (Figures 5A–5G). The few remaining GluR2 puncta at the afferent terminals of *Bai1*^*tm2b/tm2b*^ mice showed a very poor colocalization with CtBP2 (Figure 5H).

**Figure 6:**
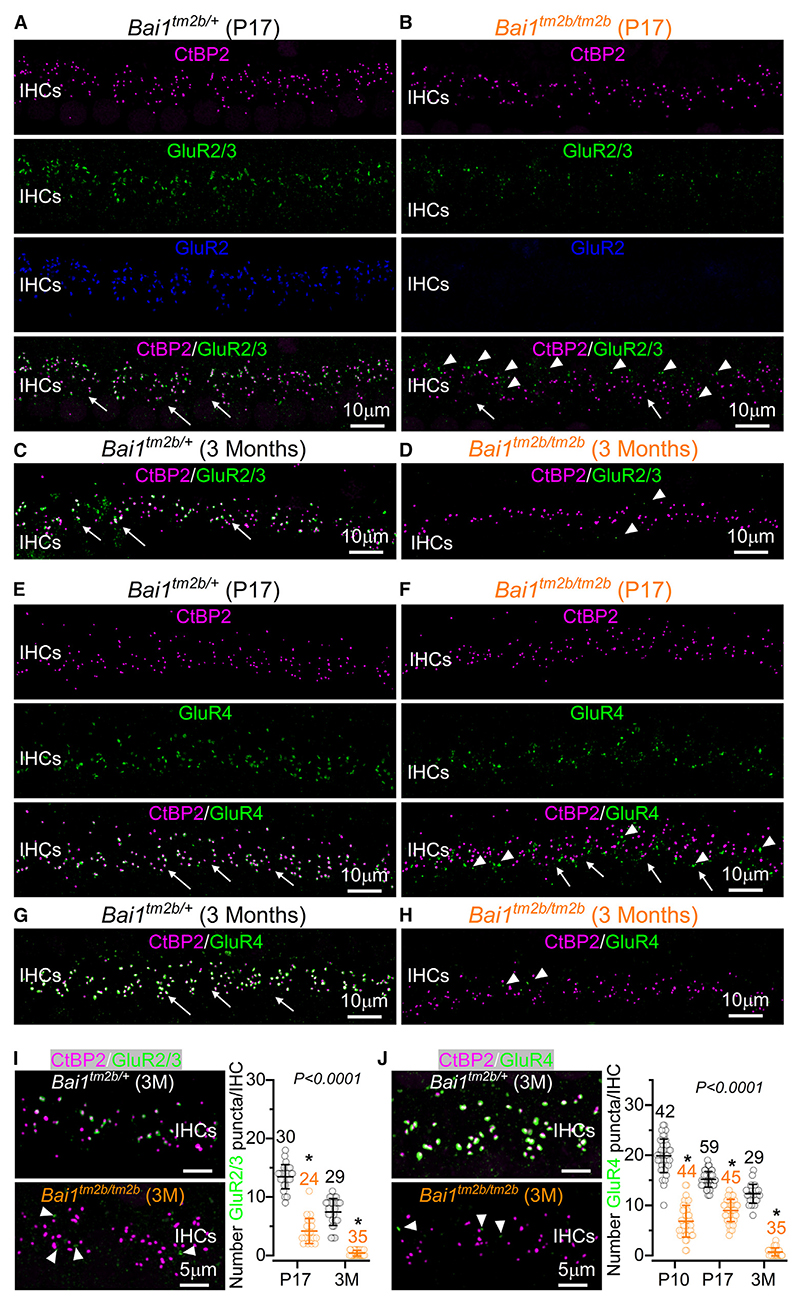
Expression of AMPA-type GluR2/3 and GluR4 receptors in the IHCs of *Bai1* mice (A–D) Maximum intensity projections of confocal z stacks of the synaptic region of the IHCs taken from the apical cochlear coil of *Bai1*^*tm2b/+*^ (A and C) and littermate *Bai1*^*tm2b/tm2b*^ (B and D) mice at P17 (A and B) and 3 months of age (C and D). IHCs were labeled with antibodies against CtBP2 (ribbon synaptic marker) and both GluR2 and GluR2/3 (post-synaptic markers). Because the only available antibody against GluR3 is also detecting GluR2 (GluR2/3), the expression of GluR3 can be identified in the IHCs from *Bai1*^*tm2b/tm2b*^, as they do not express GluR2. Arrows indicate the IHC synaptic region. The arrowheads show that GluR3 puncta are not colocalized with the CtBP2 puncta. (E–H) Images of the IHC synaptic region obtained as described above from *Bai1*^*tm2b/+*^ (E and G) and *Bai1*^*tm2b/tm2b*^ (F and H) mice at P17 and 3 months of age using antibodies against CtBP2 and GluR4 (post-synaptic marker). Arrows and arrowheads have the same meaning as described above. (I and J) Left images show enlarged synaptic areas of the IHCs highlighting the degree of colocalization between GluR2/3 (I) and GluR4 (J) and the pre-synaptic ribbon CtBP2 punctate from both genotypes. Right images show the number of GluR2/3 (I) and GluR4 (J) puncta present at the synaptic region of the IHCs from different ages. GluR2/3 (I): P17 and 3-month-old mice (3 and 2 mice, respectively, for both genotypes). GluR4 (J): P10, P17, and 3-month-old mice (*Bai1*^*tm2b/+*^: 3, 4, and 2 mice; *Bai1*^*tm2b/tm2b*^: 3, 3, and 2 mice, respectively). Numbers above the mean (±SD) data indicate the IHCs used for each genotype. Statistical values shown in (I) and (J) were obtained using Kruskal Wallis, followed by Dunn test. See also Figures S10 and S11.

GluR3 and GluR4 were still expressed in the cochlea of P7 *Bai1*^*tm2b/tm2b*^ mice, albeit to a lesser extent compared to littermate controls (Figures S10A–S10D). However, compared to control mice, the number of GluR3 (Figures 6A–6D and 6I) and GluR4 puncta (Figures 6E–6H and 6J) at the IHC synapses of *Bai1*^*tm2b/tm2b*^ mice was already reduced by 50%–70% at P10 and P17 and almost completely absent by 3 months of age. The remaining GluR3 and GluR4 puncta were largely not colocalized with the pre-synaptic ribbons at both P17 (Figures S10E–S10H) and adult mice (Figures 6I and 6J). Despite the very significant reduction of GluR4 puncta at the SGN afferent terminals of P10 *Bai1*^*tm2b/tm2b*^ mice (p < 0.0001, Tukey’s post-test, one-way ANOVA, Figure 6J), the protein level assessed with western blot was not significantly different between the two genotypes at P9–P11 (Figure S11). This indicates that although the proteins are produced, they are not localized correctly at the post-synaptic afferent terminals.

The tetrameric AMPA receptors are non-selective cation channels known to be permeable to sodium, potassium, and calcium,^43^ but any subunit combination that includes GluR2 makes them largely impermeable to calcium.^44^ Although GluR2 is ubiquitously expressed at the SGN terminals, recent evidence has indicated the presence of Ca^2+^-permeable, GluR2-lacking AMPA receptors.^45,46^ We therefore investigated whether spontaneous Ca^2+^ signals in the SGN terminals of P7–P9 *Bai1*^*tm2b/tm2b*^ mice, which represent the activation of post-synaptic receptors via the spontaneous release of glutamate from the IHCs, were affected, as they are almost completely devoid of GluR2 (Figure 5). The lack of GluR2 would increase the potential number of Ca^2+^-permeable, GluR2-lacking AMPA receptors in the SGNs of *Bai1*^*tm2b/tm2b*^ mice, thus affecting their spontaneous Ca^2+^ signals. To address this question, acutely dissected cochleae from *Bai1*^*tm2b/+*^ and *Bai1*^*tm2b/tm2b*^ mice transduced with AAV9-*syn*-GCaMP8m and -GCaMP8f at P1, a green Ca^2+^ indicator targeting SGNs, were incubated for 5 min with the red Ca^2+^ dye Rhod-2 AM that labels the IHCs. We found that spontaneous IHC depolarization elicited Ca^2+^ transients in the SGN afferent terminals of *Bai1*^*tm2b/tm2b*^ mice that were indistinguishable from littermate controls (Figure S12). These Ca^2+^ transients in SGNs were directly linked to IHC exocytosis since they were absent in mice lacking the Ca_V_1.3 Ca^2+^ channels (*Ca*_*V*_*1.3*^-/-^)^47^ (Figure S12).

### Transcriptional changes in the *Bai1^tm2b/tm2b^* mouse

**Figure 7:**
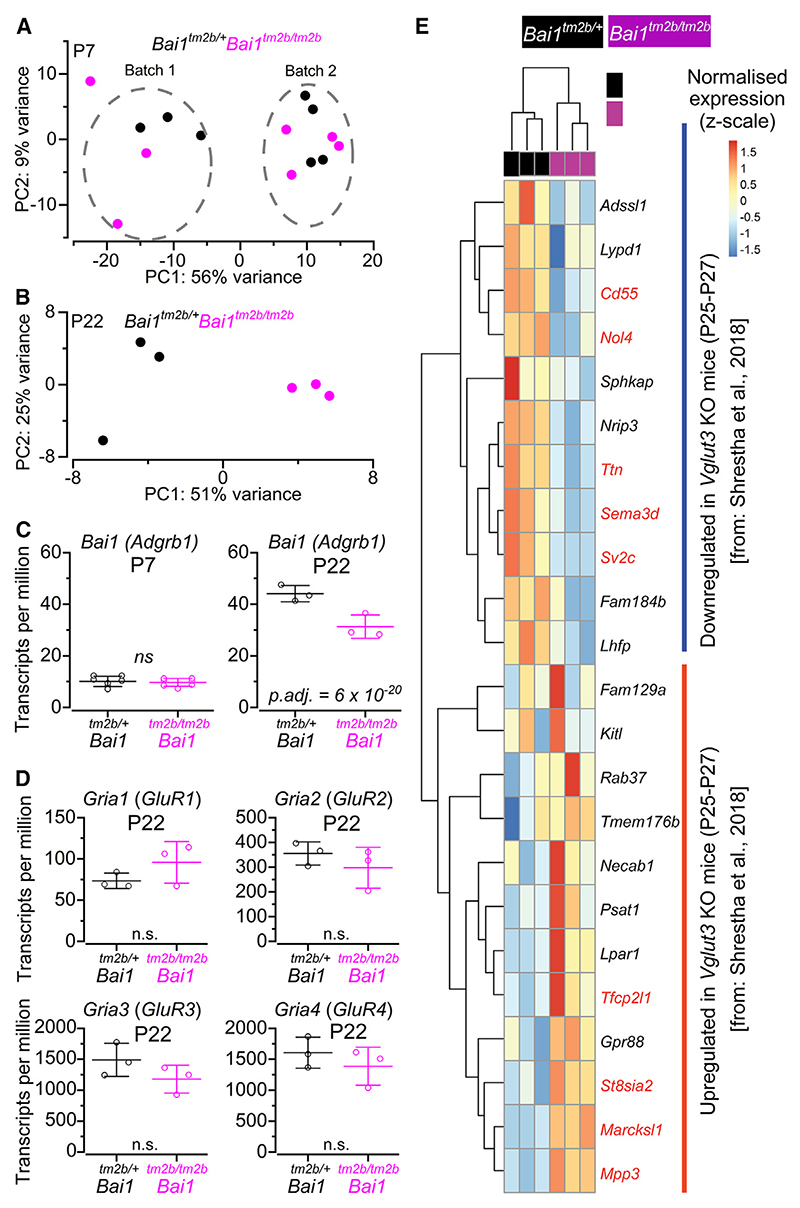
RNA sequencing analysis in *Bai1* mice (A and B) Principal-component analysis (PCA) plot of each RNA library from P7 (A) and P22 (B) control (*Bai1*^*tm2b/+*^) and *Bai1*^*tm2b/tm2b*^ mice. For P7 data, two separated batches were run from 4 (batch 1) and 3 (batch 2) samples per genotype. For P22 data, one batch was run from 3 samples per genotype. At both ages, each point represents one pool of 4 mice (8 cochleae) for both control and littermate knockout mice. Note that at P7 (A), no PC could capture genotype, consistent with the finding that no genes are differentially expressed at pre-hearing stages between control and *Bai1*^*tm2b/tm2b*^ mice. (C) Normalized counts (transcripts per million) of *Bai1* gene at P7 (left) and P22 (right) control (*Bai1*^*tm2b/+*^) and *Bai1*^*tm2b/tm2b*^ mice. Adjusted p values are based on DESEq2 analysis with a log2 fold change of 0.5 (see STAR Methods). Data are shown as mean ± SD. (D) Normalized counts (transcripts per million) of the GluR1–4 genes (*Gria1–4*). DESEq2 analysis with a log2 fold change of 0.5 (see STAR Methods) show no significant difference for any of the four *Gria* genes. (E) Heatmap of the counts (normalized, z-scale) of the genes found to be differentially expressed in *Bai1* knockout animals versus the single-cell RNA sequencing of *VGlut3* knockout SGNs.^14^ Genes that are differentially expressed in the P22 data are marked in red. Data in (C) and (D) are shown as mean ± SD. See also Figure S12.

To understand the molecular pathways underpinning the changes in the IHC synaptic machinery, we performed RNA-seq on the cochlear apical coil of P7 and P22 *Bai1*^*tm2b/+*^ and littermate *Bai1*^*tm2b/tm2b*^ mice. Surprisingly, despite observing a clear loss of the targeted exons (Figure 1F), the transcriptomes at P7 could not be clearly separated using principal-component analysis of the most variable genes. Moreover, differential gene expression analysis using DeSEQ2 did not identify any expression differences between *Bai1*^*tm2b/+*^ and *Bai1*^*tm2b/tm2b*^ mice. To determine if this was a technical issue, we repeated the P7 time point (3 replica for each time point) and again observed no change between genotypes. When both sets of data were analyzed together, principal component 1 (PC1) now captured dissection batch and experimental run (Figure 7A). Therefore, we concluded that at P7, the transcriptome of the *Bai1*^*tm2b/tm2b*^ mice was not altered.

To determine how the cochlea continues to develop postnatally in *Bai1*^*tm2b/tm2b*^ mice, we also performed RNA-seq on adult mice at P22. In contrast to P7, principal-component analysis showed a clear separation between the genotypes with PC1 explaining 51% of the observed variance (Figure 7B). By mapping reads to all isoforms of *Bai1* at P22, we found a decrease in *Bai1* expression (Figure 7C). Using splicing analysis (Figure 1F), we determined that while the short isoform was still expressed at the same level, exons from the long isoform were lost. The *Bai1* upregulation between P7 and P22 (Figure 7C) may explain the increased sensitivity of RNA-seq to detect the change.

Differential gene expression analysis from the P22 data (adjusted p value < 0.05, log2 standard deviation < 0.5, and log2 fold change > 0.5) yielded 163 upregulated genes and 241 downregulated genes (Table S1). Among the 241 downregulated genes, we observed an enrichment for genes annotated to GO processes related to potassium channels, microfilament assembly, the synaptic vesicle membrane, and the glutamate neurotransmitter release cycle (Table S2). We found that the SGN-specific GluRs GluR2–4, and GluR1, were not significantly downregulated in the *Bai1*^*tm2b/tm2b*^ mice (Figure 7D). This further supports the western blotting data showing that the level of GluR4 was not significantly different between *Bai1*^*tm2b/+*^ and *Bai1*^*tm2b/tm2b*^ mice at P9–P11 (Figure S11), despite observing a strong reduction in the number of GluR4 puncta (>50%) in the afferent terminals (Figure 6J). RNA-seq has also highlighted that NMDA receptors were not differentially expressed between the two genotypes.

Among the 163 upregulated genes, we did not observe a clear enrichment of Gene Ontology processes but did obtain significant enrichment of genes associated with hearing loss, including the upregulation of *Otof, Tectb, Cldn11, Sall1, Cldn14, Chrna10, Esprn*, and *Fgfr3* (Table S1). This upregulation suggests some compensatory mechanisms by other parts of the adult cochlea, including the hair cells. We next compared these data to the genes found to be differentially regulated in adult type I SGNs in mice lacking the vesicular glutamate transporter (VGLUT3) at the IHC synapses (*VGlut3* knockout mice).^14^ Similar to *Bai1*-deficient mice, SGN terminals are not activated in *VGlut3* knockout mice, but in this case, it is caused by the IHC’s failure to release glutamate.^48^ Of the 11 genes downregulated in the *VGlut3* knockout mice, 5 were also significantly reduced in the *Bai1*^*tm2b/tm2b*^ mice (45%), although all 11 genes showed the same overall trend (Figure 7E). Of the 12 upregulated genes, 4 were significantly changed in the *Bai1*^*tm2b/tm2b*^ mice (33%). Of note, the original VGLUT3 differentially expressed genes were determined by pseudobulked analysis of single-cell SGNs, while our analysis is based on bulk RNA extracted from cochlear tissue. Collectively, these data suggest that the compensatory changes in the adult *Bai1*^*tm2b/tm2b*^ mice phenocopy transcriptionally at least certain aspects of the *VGlut3* loss of function.

## Discussion

Here, we show that *Bai1* is expressed in the mammalian cochlea and is crucial for normal hearing. The absence of the long isoform of BAI1, which caused a failure of AMPA GluR2–4 clustering at the IHC post-synaptic afferent terminals, led to highly elevated hearing thresholds in *Bai1-*deficient mice. Cochlear RNA-seq analysis indicated that the absence of *Bai1*’s long isoform did not significantly change the gene expression profile in pre-hearing mice (P7–P10), a time when post-synaptic defects in AMPA receptor localization are already evident. However, compensatory changes of the transcriptome were present in young adult mice (P22), including gene expression changes similar to those observed in *Vglut3* loss-of-function mice.^14^ Considering that AMPA receptors are expressed at normal levels in the cochlea of *Bai1*^*tm2b/tm2b*^ mice (GluR2–4: RNA-seq) and appear to be translated into proteins (GluR4: western blotting), we propose that BAI1 is required for the correct trafficking or anchoring of GluR2–4 subunits to the PSDs. We have also found that the SGNs and their afferent fibers were still present in 7- to 8-month-old *Bai1*^*tm2b/tm2b*^ mice despite being almost completely devoid of all three GluRs, suggesting that another signal is required for the long-term survival of SGNs.

### The role of BAI1 in the mammalian cochlea

IHCs in the post-hearing mammalian cochlea have about 20 pre-synaptic ribbons^7^ that are contacted by unbranched type I SGN afferent terminals forming a large PSD.^49^ The glutamatergic PSDs at IHC ribbon synapses have a comparable morphology to those present in the CNS^50–53^ but are generally much larger.^54^ In the CNS, these PSD regions include several proteins involved in signaling, cell adhesion, cell scaffolding, the cytoskeleton, and membrane trafficking^50,55^(see also: Genes to Cognition: https://genes2cognition.org/). Although the full molecular composition of the SGN PSD is still unclear, it shares key proteins with CNS synapses such as AMPA receptors containing GluR2–4 subunits,^14,40,41^ scaffold proteins such as PSD-95, SHANK-1, and HOMER^38,56–58^ and neuroligins.^59^ BAI1 is another protein enriched in the PSD region of excitatory brain synapses^23^ and cochlear SGNs (Figure 1).

The BAI family is a subclass of adhesion G-protein-coupled receptors, and each member has seven transmembrane domains.^16^ BAI proteins possess a large and highly glycosylated N-terminal extracellular domain containing multiple thrombospondin type-1 repeats (TSRs), which have been implicated in neuronal development, including synaptogenesis, in the CNS^60–62^ and the cochlea.^63,64^ Studies in knockout mice have shown that loss of BAI1 results in hippocampal learning and memory deficits associated with abnormal synaptic plasticity and PSD thinning.^24^ BAI1, via a C-terminal PDZ binding motif, can interact directly with the PSD scaffold protein PSD-95,^65^ contributing not only to its anchoring to the post-synaptic membrane^66^ but also to its recruitment of several regulators of the actin cytoskeleton. As such, BAI1 is able to regulate dendritic spine morphogenesis and morphology.^23,67^

We have shown that cochlear SGNs express both a long and a short isoform of BAI1, but only the N terminal of the long isoform is affected in the loss-of-function *Bai1* mice (*Bai1*^*tm2b/tm2b*^). Despite the presence of the short isoform, *Bai1*^*tm2b/tm2b*^ mice exhibit almost no sound-evoked activity in the SGNs and largely reduced hearing sensitivity over the entire frequency range investigated (3–42 kHz), indicating its lack of functional compensation. This also implies that the extracellular TSR region of the long BAI1 isoform is crucial for normal hearing. The finding that ABR thresholds in *Bai1*^*tm2b/tm2b*^ mice were significantly elevated at P15, which is just a few days after the onset of hearing (P12–P13), and that the hearing phenotype does not deteriorate further with age indicate that defects have already occurred in the developing cochlea. While the morphology and function of the IHCs were unaffected in *Bai1*^*tm2b/tm2b*^ mice, the SGN afferent terminals in adult mice were almost completely devoid of all three AMPA GluRs (GluR2–4). This phenotype fully explains the greatly reduced wave 1 in the pure-tone ABR responses, which are generated by the summed response to sound of the afferent nerve fibers innervating the IHCs.^31,32^

Despite observing a clear loss of the TSRs in the long BAI1 isoform and the failed accumulation of AMPA receptors in the SGN terminals at P7 (mainly GluR2 and to a lesser extent GluR3 and GluR4), two independent RNA-seq experiments could not identify any differentially expressed genes between *Bai1*^*tm2b/+*^ and *Bai1*^*tm2b/tm2b*^ mice. These findings, together with the fact that AMPA receptors appear to be produced in *Bai1*^*tm2b/tm2b*^ co-chlea and that BAI1 is known to interact with other PSD scaffold proteins, indicate that BAI1 is most likely involved in trafficking or anchoring GluRs to the SGN post-synaptic membrane.

### Absence of functional BAI1 causes gene expression changes only in the adult cochlea

Previous findings have shown that the molecular identity of the SGN subtypes is primarily defined early on in development, most likely during embryonic stages.^13,15,68^ However, the transcriptomic specification of SGNs has been shown to undergo further refinement postnatally^14,69,70^ and depends on the spontaneous release of glutamate by the IHCs primarily during the first postnatal week.^14^ In P7 *Bai1*-deficient mice, we found that gene expression in the cochlea was indistinguishable from that of littermate control mice in two separate runs of bulk RNA-seq even though GluR2 was nearly absent and GluR3 and GluR4 somewhat reduced in the SGN afferent terminals. These findings suggest that SGN refinement in the pre-hearing cochlea could be less dependent on AMPA receptors and more reliant on other mechanisms such as, for example, the activity of NMDA receptors (see below). Despite the lack of transcriptomic variance at P7, several genes were downregulated between *Bai1*^*tm2b/+*^ and *Bai1*^*tm2b/tm2b*^ mice at P22, some of which were associated with all three subtypes of type I SGNs identified from single-cell transcriptomic analysis, including *Tnt, Rxrg, Trim54, Calb2, Obscn*, and *Cpne6*.^13–15^ These gene expression changes are likely due to downstream consequences of the lack of GluR2–4 receptors in the SGN afferent terminals of adult *Bai1*^*tm2b/tm2b*^ mice. Interestingly, several of the affected SGN genes in *Bai1*-deficient mice are also targeted in *VGlut3* knockout mice, in which IHCs can no longer release glutamate to activate the post-synaptic GluRs,^48^ suggesting that, different from pre-hearing stages, the activation of the AMPA receptors is required for the long-term maintenance of the molecular identity of the SGNs.

### Mechanism of SGN activation in the cochlea

The biophysical properties of the IHC exocytotic machines and the colocalization between PSD-95 and CtBP2 in adult *Bai1*^*tm2b/tm2b*^ mice were indistinguishable from those of control mice up to at least 8 months of age. Despite this, sound-evoked activity in *Bai1*-deficient mice devoid of AMPA receptors was near absent, as is evident from the near-zero ABR wave 1 amplitudes. These findings suggest that although GluR2–4 are crucial for sound transmission at ribbon synapses, they appear not to be required for the survival of the SGNs. However, glutamate release from the hair cells has been shown to be key not only for the function^8^ but also the survival^71^ of SGNs in the developing cochlea. For example, IHCs unable to release glutamate due to the knockdown of VGLUT3 or otoferlin, the Ca^2+^ sensor for exocytosis, lose pre-synaptic ribbons and post-synaptic afferent terminals.^48,72^ In addition to the well-defined role of AMPA-type receptors in the generation of fast excitatory post-synaptic currents in cochlear afferent terminals,^8,9,39,45^ SGNs also express NMDA receptors^73–75^ that have been shown to play a crucial neurotrophic role in their survival.^71,76^ As such, we propose that glutamate released by the IHC ribbon synapses serves to not only encode sound-induced signals via AMPA receptors but also promote the survival of SGN afferents, possibly through NMDA receptors.

### Limitations of the study

In summary, we have demonstrated that the long isoform of *Bai1* is required for establishing the functional connectivity of IHC ribbon synapses by localizing or clustering the GluRs at the post-synaptic SGN afferent terminals. The inability to determine the location of BAI1 in the cochlea (i.e., SGNs or afferent terminals) using commercially available antibodies (see STAR Methods) prevented us from determining whether BAI1 is involved in the trafficking or clustering of GluRs in the SGN afferent endings. Additionally, RNA-seq and western blot experiments indicated that AMPA receptors were most likely present in the cochlea of *Bai1*^*tm2b/tm2b*^ mice, albeit no longer clustered in the afferent terminals. The absence of GluR clustering made it extremely difficult to identify their location in the SGNs of *Bai1*^*tm2b/tm2b*^ mice.

## Star⋆Methods

### Key Resources Table

**Table:** 

REAGENT or RESOURCE	SOURCE	IDENTIFIER
Antibodies		
Anti-EPS8 Mouse-IgG1 (610143)	BD Bioscience	RRID:AB_397544
anti-Bail Rabbit-IgG (135907)	abcam	N/A
anti-Bai1 Rabbit-IgG (SAB4502506)	Sigma	RRID:AB_10747699
anti-Bai1 Rabbit-IgG (PA8-102069)	Invitrogen	N/A
Anti-Bai1 Rabbit-IgG (AP8170a)	ABCEPTA	RRID:AB_354108
Anti-ChAT Goat-IgG (AB144P)	Millipore	RRID:AB_2079751
Anti-Myo7a Rabbit-IgG (25-6790)	Proteus Biosciences	RRID:AB_10015251
Anti-Tubulin Beta 3 Mouse-IgG2a (#801201)	BioLegend	RRID:AB_2313773
Anti-Histone H3 (#9715)	Cell Signaling	RRID:AB_331563
Anti-Calretinin Rabbit-IgG (NBP1-32244)	Novus Biologicals	RRID:AB_10003923
Anti-CtBP2 Mouse-IgG1 (#612044)	Biosciences	RRID:AB_399431
Anti-PSD95 Mouse-IgG2a (MABN68)	Millipore	RRID:AB_10807979
Anti-GluR2 Mouse-IgG2a (MAB397)	Millipore	RRID:AB_2113875
Anti-GluR2/3 Rabbit-IgG (AB1506)	Millipore	RRID:AB_90710
Anti-GluR4 Rabbit-IgG (#8070)	Cell Signaling	RRID:AB_10829469
Anti-Shank1a Rabbit-IgG (RA19016)	Neuromics	RRID:AB_1622814
Donkey Anti-Goat-IgG NL556 (NL001)	RNDSystems	RRID:AB_663766
Goat Anti-Rabbit-IgG Alexa Fluor 405 (A31556)	ThermoFisher	RRID:AB_221605
Goat Anti-Mouse-IgG1 Alexa Fluor 647 (A21240)	ThermoFisher	RRID:AB_2535809
Goat Anti-Mouse-IgG2a Alexa Fluor 488 (A21131)	ThermoFisher	RRID:AB_2535771
Goat Anti-Rabbit-IgG Alexa Fluor 647 (A21245)	ThermoFisher	RRID:AB_2535813
Goat Anti-Rabbit-IgG Alexa Fluor 488 (A11034)	ThermoFisher	RRID:AB_2758380
Anti-GAPDH Mouse-IgG HRP (#9484)	Abcam	RRID:AB_307274
Goat Anti-Mouse-IgG2a HRP (#A-10685)	ThermoFisher	RRID:AB_2534065
Sheep Anti-Mouse-IgG HRP (#NA931)	Cytiva	RRID:AB_772210
Donkey Anti-Rabbit-IgG HRP (#NA934)	Cytiva	RRID:AB_772206
Goat Anti-Rabbit IgG HRP (#31460)	Invitrogen	RRID:AB_228341
Bacterial and virus strains		
pGP-AAV-*syn*-jGCaMP8m-WPRE	Addgene	Addgene viral prep_162375-AAVrg
pGP-AAV-*syn*-jGCaMP8f-WPRE	Addgene	Addgene viral prep_162376-AAVrg
Chemicals, peptides, and recombinant proteins		
Texas Red-X Phalloidin	ThermoFisher	T7471
VectaShield	Vector Laboratories	H1000
ECL Prime Western Blotting Reagent	Cytiva	#RPN2232
Pierce™ RIPA Buffer	Thermo Scientific	89901
Critical commercial assays		
RNeasy Plus Micro Kit	Qiagen	74034
Deposited data		
RNA-sequencing data	Gene Expression Omnibus (GEO)	GSE254269
Experimental models: Organisms/strains		
*Adgrb^tm2a^* allele	MRC Harwell Institute	EM:08738
Oligonucleotides		
genotyping primer forward WT: 5′ CCAGTTGGTCTGGTGTCA 3′	ThermoFisher	Customize
genotyping primer forward LacZ: 5′ CAGACCCAGACCTTG AGGAG 3′	ThermoFisher	Customize
genotyping primer reverse WT: 5′ CGCAGGTACTGGAGCA TACA 3′	ThermoFisher	Customize
qPCR primer forward mm_Hsp90b1: 5′ GAGTCTCCCTG TGCTCTTGT 3′	ThermoFisher	Customize
qPCR primer reverse mm_Hsp90b1: 5′ CATCTTCCTTAA TCCGCCGC 3′	ThermoFisher	Customize
qPCR primer forward mm_Aco1: 5′ CCGGGATGTTTAAG GAGGT3′	ThermoFisher	Customize
qPCR primer reverse mm_Aco1: 5′ GGCTGGAGATCTAAA GTCAAGC 3′	ThermoFisher	Customize
qPCR primer forward mm_Adgrb1: 5′ CATGCGGCTGAGA AGGAGAA 3′	ThermoFisher	Customize
qPCR primer reverse 5′ CCTCTTGTTGGGAGTCTGCT 3′	ThermoFisher	Customize
Software and algorithms		
Origin Microcal	OriginLab	RRID:SCR_002815
GraphPad Prism	GraphPad	RRID:SCR_002798
ImageJ Fiji	ImageJ	RRID:SCR_002285
BioSigRZ	Tucker-Davis Technologies	RRID:SCR_014820
pClamp	Molecular Devices	RRID:SCR_011323
ImageLab	Bio-Rad	RRID:SCR_014210
Python 3.7	Python	RRID:SCR_008394
R Project	The R Foundation	RRID:SCR_001905
nf-core:	https://github.com/nf-core/rnaseq	N/A
Other		
Agilent Tapestation 4200	Agilent	RRID:SCR_019394
Reichert Jung Ultracut E Ultramicrotome	Reichert Jung	RRID:SCR_022980
Transmission Electron Microscope	JEOL	JEOL 100S
35mm Acros Neopan Film	Fujifilm	ACROS 100 II
Canonscan Negative Scanner	Canon	Canonscan 9000F
Cryostat	ThermoFisher	CryoStar NX70

### Resource Availability

#### Lead contact

Further information and requests for resources and reagents should be directed to and will be fulfilled by the lead contact, Walter Marcotti (w.marcotti@sheffield.ac.uk).

#### Materials availability

No reagents or materials were generated from this study.

### Experimental Model and Study Participant Details

#### Animal model

The *Adgrb1*^*tm2a*^ allele (EM:08738) was imported from the EMMA repository at the University of Veterinary Medicine, Austria, to the MRC Harwell Institute (UK) and licensed by the Home Office under the Animals (Scientific Procedures) Act 1986 (PPL_PBF9BD884) and approved by the local Ethical Review Board (AWERB). To obtain *Adgrb1*^*tm2b*^ knockout mice, cre-mediated conversion of the ‘knockout-first’ tm1a allele was achieved by treating IVF derived embryos with a cell permeable cre-enzyme (Excellgen). Because *Adgrb1*^*tm2b*^ mice encode for the protein Brain-Specific Angiogenesis Inhibitor 1 (Bai1), we named the mice *Bai1*^*tm2b*^. These mice were generated and maintained on the C57BL/6N background strain. The *Bai1*^*tm2b*^ mice are viable and fertile, and the frequency of homozygous and heterozygous offspring follow the expected Mendelian ratio. Mice used for this study and had free access to food and water and a 12 h light/dark cycle.

Both male and female mice ranging from postnatal day 6 (P6) and P288 were used for this study.

Mice were genotyped by extracting their DNA from the tissue of ear- or tail-clips, which was used as the template for PCR using the following primers: forward WT: 5′ CCA GTT GGT CTG GTG TCA 3; forward LacZ:5′ CAG ACC CAG ACC TTG AGG AG 3’; reverse WT: 5′ CGC AGG TAC TGG AGC ATA CA 3’.

### Method Details

#### Ethics statement

The animal work was licensed by the UK Home Office under the Animals (Scientific Procedures) Act 1986 (PPL_PCC8E5E93) and was approved by the University of Sheffield Ethical Review Committee (180626_Mar). For *in vitro* experiments mice were killed by cervical dislocation followed by decapitation. For *in vivo* auditory brainstem responses (ABRs) and distortion product otoacoustic emissions (DPOAEs) mice were anesthetized using intraperitoneal injection of ketamine (100 mg/kg body weight, Fort Dodge Animal Health, Fort Dodge, USA) and xylazine (10 mg/kg, Rompun 2%, Bayer HealthCare LLC, NY, USA). Following the onset of anesthesia and the loss of the retraction reflex with a toe pinch, mice were placed in a soundproof chamber for *in vivo* experiments. At the end of the *in vivo* recordings, mice were either culled by cervical dislocation or recovered from anesthesia with intraperitoneal injection of atipamezole (1 mg/kg). For *in vivo* gene-delivery, mice were anesthetized with isoflurane (2.5%) under oxygenation (0.8%). Mice under recovery from anesthesia were returned to their cage, placed on a thermal mat and monitored over the following 2–5 h.

#### Tissue preparation

The cochlea was dissected out from both male and female mice in an extracellular solution composed of (in mM): 135 NaCl, 5.8 KCl, 1.3 CaCl_2_, 0.9 MgCl_2_, 0.7 NaH_2_PO_4_, 5.6 D-glucose, 10 HEPES-NaOH. Amino acids, vitamins and sodium pyruvate (2 mM) were added from concentrates (Thermo Fisher Scientific, UK). The pH was adjusted to 7.48 with 1M NaOH (osmolality ~308 mOsm kg^–1^). The dissected cochleae were transferred to a microscope chamber and immobilised via a nylon mesh attached to a stainless-steel ring. The microscope chamber, which was continuously perfused with the above extracellular solution using a peristaltic pump (Cole-Palmer, UK), was then mounted on the stage of an upright microscopes (Olympus BX51, Japan; Leica DMLFS, Germany) with Nomarski Differential Interference Contrast (DIC) optics (60x or 64x water immersion objective) and a 15× eyepiece.

#### Auditory brainstem responses

Anesthetized mice were placed in a soundproof chamber (MAC-3 acoustic chamber, IAC Acoustic, UK). Male and female mice were placed on a heated mat (37°C) with the animal’s pinna positioned at 10 cm from the loudspeaker (MF1-S, Multi Field Speaker, Tucker-Davis Technologies, USA), which was calibrated with a low-noise microphone probe system (ER10B+, Etymotic, USA). Two subdermal electrodes were placed under the skin behind the pinna of each ear (reference and ground electrode), and one electrode half-way between the two pinna on the vertex of the cranium (active electrode). Experiments were performed using a customized software^77,78^ driving an RZ6 auditory processor (Tucker-Davis Technologies). ABR responses were measured for white noise clicks and pure tone stimuli of frequencies at 3, 6, 12, 18, 24, 30 and 36 kHz. ABR thresholds were defined as the lowest sound level where any recognisable feature of the waveform was visible. Stimulus sound pressure levels were up to 95 dB SPL, presented in steps of 5 dB SPL (average of 256 repetitions). Tone bursts were 5 ms in duration with a 1 ms on/off ramp time presented at a rate of 42.6/s.

Wave 1 amplitude and latency were measured from ABR recordings obtained by stimulating mice with a pure tone (12 kHz). We selected the 12 kHz value as it is close to the frequency range used for the *in vitro* work. An initial automatic identification of Wave 1 was carried out using a custom software routine based on the *find_peaks* function of the scipy.signal Python module (Python 3.7, Python software foundation).^79^ Results were manually reviewed and, if required, adjusted to the correct peak. The Wave 1 amplitude was calculated as the difference between the amplitude of the first peak and the first trough of the ABR waveform; the latency was calculated as the delay of the Wave 1 peak from the beginning of the recording. Since the distance of the speaker from the animal is 10 cm (see above), this leads to a delay in the signal of ~0.3 ms.

#### Distortion product otoacoustic emissions

Distortion product otoacoustic emissions (DPOAEs) were used to assess the function of OHCs by the synchronous presentation of two stimulus tones (primaries f1 and f2). DPOAEs were recorded at 2f1-f2 in response to primary tones f1 and f2, where f2/f1 = 1.2. The f2 level (L2) was set from 20 to 80 dB (maximum level set for our system) in 10 dB increments, and the f1 level (L1) was set equal to L2. Frequency pairs of tones between f2 = 6.5 kHz and f2 = 26.3 kHz were presented directly into the left ear canal of mice by means of a coupler, which was connected to two calibrated loudspeakers using 3 cm plastic tubes (MF1-S, Multi Field Speaker, Tucker-Davis Technologies, USA).

Recordings were performed in a soundproof chamber (MAC-3 Acoustic Chamber, IAC Acoustic, UK) and the emission signals were recorded by a low-noise microphone (ER10B+: Etymotic Research Inc, USA) connected to the coupler mentioned above. Experiments were performed using BioSigRZ software driving an RZ6 auditory processor (Tucker-Davis Technologies). The DPOAE thresholds were defined by the minimal sound level where the DPOAEs were above the standard deviation of the noise. The determined DPOAE thresholds were plotted against the geometric mean frequency of f1 and f2. Stimulus sound pressure levels were up to 80 dB SPL, presented in steps of 10 dB. The response signal was averaged over 500 repetitions.

#### Whole-cell electrophysiology

Patch clamp recordings were performed from hair cells positioned at the apical coil region (9–12 kHz) of the cochlea. Recordings were performed at room temperature (20°C–24°C) using an Optopatch amplifier (Cairn Research Ltd, UK). Patch pipettes were pulled from soda glass capillaries, which had a typical resistance in extracellular solution of 2–3 MΩ. The intracellular solution used for the patch pipette contained (in mM): 131 KCl, 3 MgCl_2_, 1 EGTA-KOH, 5 Na_2_ATP, 5 HEPES-KOH, 10 Na-phosphocreatine (pH was adjusted with 1M KOH to 7.28; 294 mOsm kg^–1^). Data acquisition was controlled by pClamp software using a Digidata 1440A (Molecular Devices, USA). In order to reduce the electrode capacitance, patch electrodes were coated with surf wax (Mr Zoggs SexWax, USA). Recordings were low-pass filtered at 2.5 kHz (8-pole Bessel), sampled at 5 kHz and stored on a computer for offline analysis (Clampfit, Molecular Devices; Origin 2021: OriginLab, USA). Membrane potentials under voltage-clamp conditions were corrected offline for the residual series resistance *R*_s_, which was normally compensated by 80%, and the liquid junction potential (LJP) of –4 mV, which was measured between electrode and bath solutions.

To investigate the biophysical characteristics of the mechanoelectrical transducer (MET) current, we displaced the hair bundles using a fluid-jet system from a pipette driven by a 25 mm diameter piezoelectric disc.^80,81^ The pipette was pulled from borosilicate glass to a final overall length of 5.5 cm. The fluid jet pipette tip had a diameter of 8–10 μm and was positioned near the hair bundles to elicit a maximal MET current (typically 10 μm). Patch pipettes contained (in mM): 135 CsCl, 2.5 MgCl2, 1 EGTA-CsOH, 2.5 Na2ATP, 10 sodium phosphocreatine, 5 Hepes-CsOH (pH 7.3). Membrane potentials were corrected offline for the LJP of –4 mV. Mechanical stimuli were applied as 50 Hz sinusoids (filtered at 1 kHz, 8-pole Bessel). Prior to the positioning of the fluid jet by the hair bundles, any steady-state pressure was removed by monitoring the movement of debris in front of the pipette.

Real-time changes in membrane capacitance (Δ*C*_m_) were measured using the track-in circuitry of the Optopatch amplifier.^36^ A4 kHz sine wave of 13 mV RMS was applied to IHCs from –81 m and was interrupted for the duration of the voltage step. The capacitance signal from the Optopatch was amplified (×50), filtered at 250 Hz and sampled at 5 kHz Δ*C*_m_ was measured by averaging the Cm trace over a 200 ms period following the voltage step and subtracting the pre-pulse baseline. Data were acquired using pClamp software and a Digidata 1440A (Molecular Devices) and analyzed with Origin (OriginLab). The intracellular solution used for the patch pipette contained (in mM): 106 Cs-glutamate, 20 CsCl, 3 MgCl2, 1 EGTA-CsOH, 5 Na2ATP, 0.3 Na2GTP, 5 HEPES-CsOH, 10 Na2-phosphocreatine (pH 7.3, 294 mOsm kg^–1^). Δ*C*_m_ was recorded in the presence of K^+^ channel blockers TEA (30 mM), 4-AP (15mM) and linopirdine (80 μM) in the extracellular solution. Membrane potentials were corrected for the voltage drop across the series resistance and an LJP of –11 mV.

#### Two-photon confocal Ca^2+^ imaging

Acutely dissected cochleae from *Bai1* mice transduced with AAV9-GCaMP8m (see below) were incubated for 5 min at RT in extra-cellular solution supplemented with Rhod-2 a.m. at a final concentration of 10 μM (#R1244, ThermoFisher Scientific, UK). The incubation medium contained also pluronic F-127 (0.1%, w/v) and sulfinpyrazone (250 lM) to prevent dye sequestration and secretion. Imaging was performed using a two-photon laser-scanning microscope^80,82^(Bergamo II System B232, Thorlabs Inc., USA) based on a mode-locked laser system operating at 800 nm, 80-MHz pulse repetition rate, <100-fs pulse width (Mai Tai HP DeepSee, Spectra-Physics, USA). Images were captured with a 60x objective (LUMFLN60XW, Olympus, Japan) using a GaAsp PMT (Hamamatsu) coupled with a 525/40 band-pass filter (FF02-525/40-25, Semrock). Images were analyzed offline using custom built software routines written in Python (Python 3.10, Python Software Foundation) and ImageJ (NIH). Calcium signals were measured as relative changes of fluorescence emission intensity (Δ*F*/*F*_0_). The correlation coefficient was calculated in a time window of 10 s centered on the maximal response of the fibers. The traces have been corrected with a rolling average filter of 500 frames before calculation of correlations. To perform the statistical test (see below), we have converted the coefficients using Fisher’s transformation.

#### AAV gene delivery in mice

The surgical protocol used for AAV injection into the cochlea of P1-P3 *Bai1* mice was performed under anesthesia. The right ear was accessed via an incision just below the pinna. After the gentle separation of the cervical muscles with forceps, the otic bulla was exposed and opened to visualize the stapedial artery and the round window membrane (RWM).^83^ When the RWM was identified, it was gently punctured with a borosilicate pipette. This was followed by the injection of the AAV into the cochlea (pressure controlled by mouth) of 1 μL of AAV9-jGCaMP8m (pGP-AAV-*syn*-jGCaMP8m-WPRE, #162375, Addgene, USA) and AAV9-jGCaMP8f (pGP-AAV-*syn*-jGCaMP8f-WPRE, #162376, Addgene, USA) at 2 × 10^13^ vg/ml. Following the injection, the pipette was retracted from the RWM and the wound was closed with veterinarian glue.

#### Scanning electron microscopy (SEM)

After dissecting out the inner ear from the mouse, the cochlea was gently perfused with fixative for 1–2 min through the round window using a 10 μL pipette tip. A small hole in the apical portion of cochlear bone was made prior to perfusion to allow the fixative to flow out from the cochlea. The fixative contained 2.5% v/v glutaraldehyde in 0.1M sodium cacodylate buffer plus 2 mM CaCl_2_ (pH 7.4). The inner ears were then immersed in the above fixative and placed on rotating shaker for 2 h at room temperature. After the fixation, the organ of Corti was exposed by removing the bone from the apical coil of the cochlea and then immersed in 1% osmium tetroxide in 0.1 M cacodylate buffer for 1 h. For osmium impregnation, which avoids gold coating, cochleae were incubated in solutions of saturated aqueous thiocarbohydrazide (20 min) alternating with 1% osmium tetroxide in buffer (2 h) twice (the OTOTO technique).^84^ The cochleae were then dehydrated through an ethanol series and critical point dried using CO_2_ as the transitional fluid (Leica EM CPD300) and mounted on specimen stubs using conductive silver paint (Agar Scientific, Stansted, UK). The apical coil of the organ of Corti was examined at 10 kV using a Tescan Vega3 LMU scanning electron microscope. For SEM, 3 mice were processed for each genotype. Images were taken from the same region (around 12 kHz) used for the electrophysiological recordings.

#### Transmission electron microscopy (TEM)

For TEM cochleae were fixed as for SEM but postfixed by immersion for 1 h in 1% osmium tetroxide in 0.1M cacodylate buffer, dehydrated and embedded in Spurr resin.^85^ Ultrathin sections (70–100 nm) were cut in radial planes from the apical coil using a Reichert ultracut E ultramicrotome, mounted on 200 mesh thin bar copper grids (Agar Scientific, Stansted, UK) and stained with 2% uranyl acetate in 70% ethanol for 20 min, followed by 2% lead citrate dissolved at high pH in distilled water for 5 min. Samples were examined in a JEOL 100S electron microscope operated at 100 kV accelerating voltage. Digital images were acquired in using a 35 mm Acros Neopan film which, once developed, was digitised using a Canonscan 9000F negative scanner. For TEM, 3 mice were processed for each genotype. Images were taken from the same region (around 12 kHz) used for the electrophysiological recordings.

#### Immunofluorescence microscopy

For pre-hearing mice, the inner ear was dissected out and immersed for 20 min at room temperature in a solution containing 4% paraformaldehyde in phosphate-buffered saline (PBS, pH 7.4). For adult mice, the inner ear was initially gently perfused with the above solution for 1–2 min through the round window. Following this initial brief fixation, the inner ear was fixed for a further 20 min at room temperature. Fixed inner ears were then washed three times in PBS for 10 min and the cochleae dissected out using fine forceps and incubated in PBS supplemented with 5% normal goat or horse serum and 0.5% Triton X-100 for 1 h at room temperature. The samples were immunolabelled with primary antibodies overnight at 37°C, washed three times with PBS and incubated with the secondary antibodies for 1 h at 37°C. Antibodies were prepared in 1% serum and 0.5% Triton X-100 in PBS. Primary antibodies were: mouse-IgG1 anti-Eps8 (1:1000, BD Biosciences, 610143), goat-IgG anti-ChAT (1:500, Millipore, AB144P), rabbit-IgG anti-MYO7a (1:500, Proteus Biosciences, 25–6790), mouse IgG2a anti-β-tubulin (1:400, BioLegend, #801201), mouse IgG1anti-CtBP2 (1:200, Biosciences, #612044), mouse-IgG2a anti-PSD95 (1:1000, Millipore, MABN68), mouse IgG2a anti-GluR2 (1:200, Millipore, MAB397), rabbit-IgG anti-GluR2/3 (1:200, Millipore, AB1506), rabbit-IgG anti-GluR4 (1:500, Cell Signaling, #8070) and rabbit-IgG anti-Shank1a (1:1000, Neuromics, RA19016). F-actin was stained with Texas Red-X phalloidin (1:400, ThermoFisher, T7471) within the secondary antibody solution. Secondary antibodies were species appropriate Alexa Fluor or Northern Lights secondary antibodies. Samples were mounted in VECTASHIELD (H-1000). The images from the apical cochlear region (around 12 kHz) were captured with Nikon A1 confocal microscope equipped with Nikon CFI Plan Apo 60x Oil objective or a Zeiss LSM 880 AiryScan equipped with Plan-Apochromat 63x Oil DIC M27 objective for super-resolution images of hair bundles. Both microscopes are part of the Wolfson Light Microscope Facility at the University of Sheffield. Image stacks were processed with Fiji ImageJ software. At least 3 mice for each genotype were used for each experiment.

#### X-gal staining

In the conditional-ready design used to generate the *Adgrb1* mutant allele, a LacZ trapping cassette gene was inserted into intron 2 placing it under the control of the *Adgrb1* promoter, allowing the visualisation of *Adgrb1* expression using X-gal. The cochlea from 6 wild-type and 6 heterozygous littermates at P6-P7 were dissected out from the inner ear and fixed with 4% paraformaldehyde for 45 min at 4°C, followed by three 10 min PBS washes with rocking. The apical spiral of the cochlea was then dissected carefully to preserve the spiral ganglion neuronal cell bodies within the modiolus before being washed for 30 min with detergent solution containing 2 mM MgCl2, 0.02% NP-40 substitute (Roche #11754599001) and 0.1% sodium deoxycholate in PBS (Oxoid #BR0014G). To produce staining solution, X-gal (Promega #V3941) was added 1:50 to 100 μL/cochlea of pre-warmed staining solution containing 5 mM K_3_Fe(CN)_6_(III) and 5mM K_4_Fe(CN)_6_(II) in detergent solution. Immediately after X-gal dilution, cochleae were incubated in darkness in staining solution overnight at 37°C, followed by two 5 min washes in PBS with rocking. Cochleae were mounted on slides and imaged using the Leica M16 microscope equipped with a 2.0x Apocromatic Corr objective and a color camera (DFC295). Images were taken using LAS-X software (Leica).

#### Western blot

To obtain protein lysates for SDS-PAGE, both cochleae from one or two animals were dissected in sterile PBS to remove the vestibular system, the bone surrounding the cochlea and the stria vascularis before being flash-frozen in liquid nitrogen, Cochleae were then crushed with a sterilised plastic pestle in 100 μL of RIPA buffer (Pierce) with 1x protease inhibitor cocktail (Roche #11836153001). Samples were vortexed every 10 min and incubated on ice for a total of 30 min followed by centrifugation at 12000 x *g* for 30 min at 4°C. Supernatants were then collected and stored at –20°C until being run on a 4–15% SDS-PAGE gel (Bio-Rad #4561083). Following 30V overnight transfer onto a PVDF membrane at 4°C, blots were blocked with 5% low fat skimmed milk powder in TBST (20 mM Tris, 150 mM NaCl, 0.1% Tween 20, pH 7.4) for 1 h at room temperature. The blot was then incubated with primary antibodies (anti-GluR2 1:1000, Millipore, MAB397; anti-GluR4 1:400, Cell Signaling, #8070; anti-GAPDH 1:1000, Proteintech, #9484; anti-class III beta-Tubulin 1:1000, BioLegend, 801201; anti-Histone H3 1:1000, CellSignalling, #9715) diluted in blocking buffer overnight at 4°C, rinsed three times and washed three times with TBST for 10 min, and then incubated with secondary antibodies (anti-mouse IgG2a 1:1000, Invitrogen, #A-10685; anti-mouse IgG 1:6000, Cytiva, #NA931; anti-rabbit IgG 1:3000, Cytiva, #NA934; anti-rabbit IgG 1:5000, Invitrogen, #31460) for 2 h at room temperature. Following three rinses and three 10 min washes with TBST, blots were developed with ECL primer western blotting reagent (Cytiva #RPN2232) and imaged on a Gel-Doc XR + system. Images were captured and analyzed using ImageLab software.

#### qPCR gene expression analysis

The apical coil of the cochlea was snap frozen after dissection and then thawed on ice in preparation for RNA extraction. Both cochleae from 3 to 4 mice were combined in one tube. Tissues were homogenized in 350ul of RLT buffer + DTT using a pestle until the tissue was no longer visible. The homogenized lysate was mixed with 350 μl of 70% ethanol and then applied directly to the Qiagen RNeasy Micro Kit according to the manufacturer’s instructions and RNA was eluted into 15 μl of dH20. ~200 ng was used for reverse transcription after nanodrop quantification using the Applied Biosystems High-Capacity RNA-to-cDNA kit. Primers were designed targeting different *Bai1* isoforms (*Adgrb*) with *Hsp90b1* and *Aco1* as housekeeping controls. Real Time-quantitative PCR was performed using the Applied Biosystems PowerUp SYBR Green Master Mix, according to the manufacturer’s instructions using the Applied Biosystems QuantStudio 12k Flex machine. Relative gene expression was calculated using the delta-delta Ct method using *Hsp90b1* as the reference and confirmed with *Aco1*. Only *Hsp90b1* is plotted the graphs that were presented. The qPCR primer sequences are the following: forward mm_Hsp90b1: 5′ GAGTCTCCCTGTGCTCTTGT 3; reverse mm_Hsp90b1: 5′ CATCTTCCTTAATCCGCCGC 3; forward mm_Aco1: 5′ CCCGGGATGTTTAAGGAGGT 3; reverse mm_Aco1: 5′ GGCTGGAGATCTAAAGTCAAGC 3; 3; forward mm_Adgrb1: 5′ CATGCGGCTGAGAAGGAGAA 3; reverse mm_Adgrb1: 5′ CCTCTTGTTGGGAGTCTGCT 3.

#### RNA isolation and library preparation for RNA-sequencing

The sensory epithelium and spiral ganglion neurons from 4 mice were micro dissected in DNase free ice-cold PBS 1X and immediately snap frozen in liquid nitrogen. Two batches of 3 and 4 replicates (P7) and one batch of 3 replicates (P22) from each genotype. RNA was extracted using RNeasy Plus Micro Kit (Qiagen) according to manufacturer’s instructions. RNA quantity was established using a Nanodrop spectrophotometer and RNA integrity number (RIN) was calculating using a BioAnalyzer (Agilent Tapestation 4200). All samples had RIN score greater than 9.1. mRNA library preparation was performed using poly A enrichment and sequenced on the Illumina NovaSeq sequencer using paired-end 150bp reads.

#### RNA-sequencing analysis and differential gene expression

The sequencing libraries were processed using the nf-core RNA pipeline^86^ (https://nf-co.re/rnaseq/usage) using the standard parameters. Reads were mapped to the mouse genome (mm10). The resulting gene counts were determined using Salmon^87^ and used for downstream analysis with DeSeq2.^88^ Metascape^89^ and Reactome^90^ were used to query for enriched GO and pathways in the list of differentially expressed genes. RPM (reads per million) bigwig files were created using R using the packages Rsamtools,^91^ rtracklayer,^92^ and Genomic Ranges^93^ and were visualized using the Washington University Genome browser (http://epigenomegateway.wustl.edu/).

### Quantification and Statistical Analysis

Statistical comparisons of means were made by Student’s two-tailed *t* test or Mann–Whitney U test (when normal distribution could not be assumed), for multiple comparisons, analysis of variance (one- or two-way or two-way ANOVA followed by a suitable post-test) or Kruskal Wallis, followed by Dunn test. p < 0.05 was selected as the criterion for statistical significance. Only mean values with a similar variance between groups were compared. Average values are quoted in text and figures as means ± S.D. Animals of either sex were randomly assigned to the different experimental groups. No statistical methods were used to define sample size, which was defined based on previous published similar work from our laboratory. Animals were taken from several cages and breeding pairs over a period of several months. Most of the electrophysiological and morphological (but not imaging) experiments were performed blind to animal genotyping and in most cases, experiments were replicated at least 3 times.

## Supplementary Material

Differential gene expression analysis from the P22 RNA sequencing data

Figures S1–S12 and Table S2

## Data Availability

Raw RNA-sequencing files are deposited on GEO under accession number: GSE254269. This paper does not report original code. Any additional information required to reanalyze the data reported in this work paper is available from the lead contact upon request.

## Highlights

- In the mouse cochlea, BAI1 is expressed in the afferent spiral ganglion neurons (SGNs)
- AMPA receptors are absent in the SGN afferent terminals of adult *Bai1*-deficient mice
- Functional IHC ribbon synapses fail to relay sound information to SGNs in mice lacking BAI1
- *Bai1*-deficient mice have highly elevated hearing thresholds

## In brief

Carlton et al. show that BAI1 is essential for activating SGN afferent terminals at cochlear IHC ribbon synapses. In *Bai1*-deficient mice, IHC-SGN synapses are formed but AMPA receptors that are normally in the post-synaptic density are either missing or uncoupled from the pre-synaptic ribbons, leading to highly raised hearing thresholds.
