# Supplementary material for: BAI1 localizes AMPA receptors at the cochlear afferent post-synaptic density and is essential for hearing
Source: Cell Rep. Author manuscript; Available in PMC 2025 Nov 20. (PMC7618386; doi:10.1016/j.celrep.2024.114025)
Supplement: Figures S1–S12 and Table S2 [file EMS210735-supplement-Figures_S1_S12_and_Table_S2.pdf]

Cell Reports, Volume *43*

Supplemental information

BAI1 localizes AMPA receptors

at the cochlear afferent post-synaptic density

and is essential for hearing

Adam J. Carlton, Jing-Yi Jeng, Fiorella C. Grandi, Francesca De Faveri, Ana E. Amariutei, Lara De Tomasi, Andrew O'Connor, Stuart L. Johnson, David N. Furness, Steve D.M. Brown, Federico Ceriani, Michael R. Bowl, Mirna Mustapha, and Walter Marcotti

BAI1 localises AMPA receptors at the cochlear afferent postsynaptic density and is essential for hearing

Adam J. Carlton, Jing-Yi Jeng, Fiorella C. Grandi, Francesca De Faveri, Ana Amariutei, Lara De Tomasi, Andrew O'Connor, Stuart L. Johnson, David N. Furness, Steve D. M. Brown, Michael R. Bowl, Mirna Mustapha, Federico Ceriani, Walter Marcotti

This document includes:

Supplementary Figures 1-12

Supplementary Table 2.

Figure 1

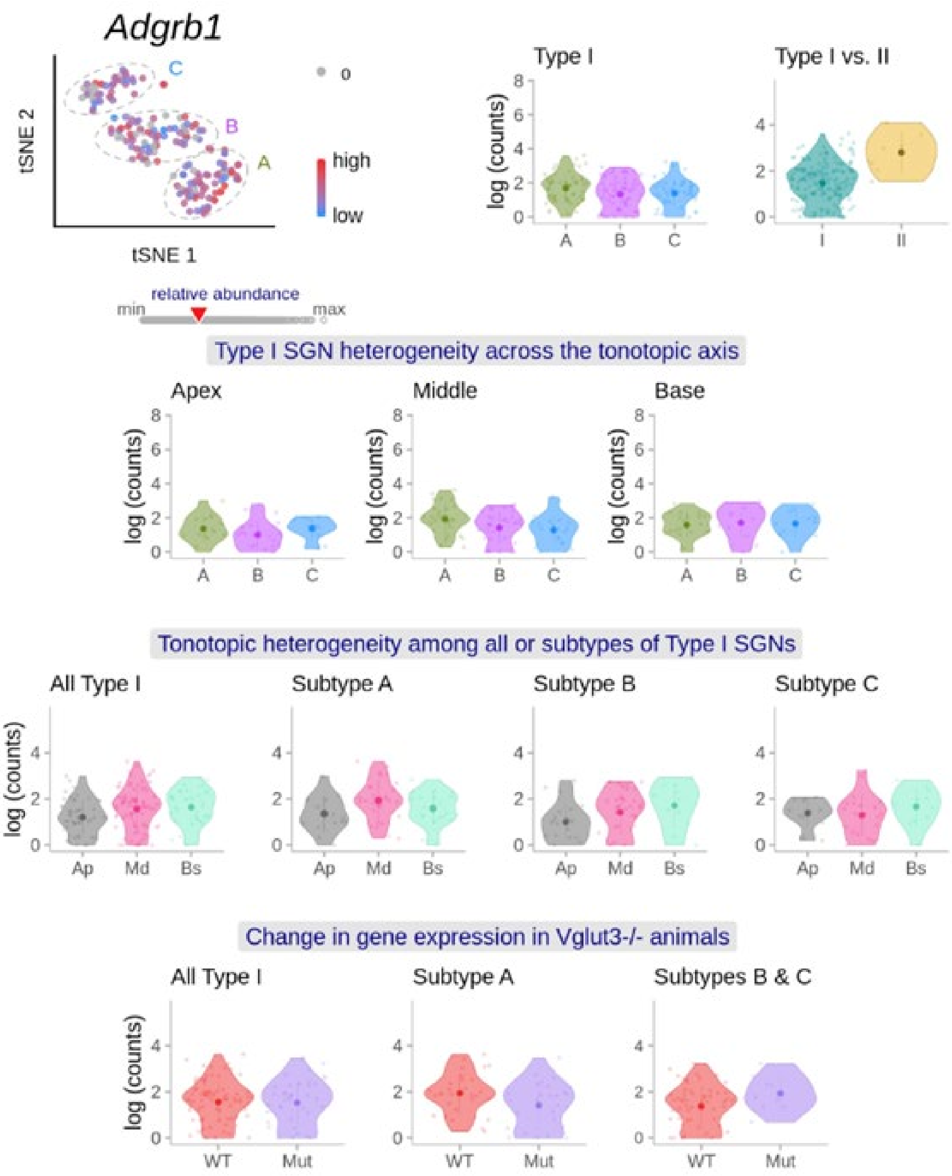

Supplementary Figure 1: All SGNs exhibit expression of *Bai1* (*Adgrb1*). Related to Figure 1. Violin plots comparing the expression of *Bai1* (*Adgrb1*) among types I and II SGNs. Data are from: Shrestha et al., 2018.

Shrestha BR, Chia C, Wu L, Kujawa SG, Liberman MC, Goodrich LV (2018). Sensory Neuron Diversity in the Inner Ear Is Shaped by Activity. Cell 174:1229-1246.e17.

Supplementary Figure 2

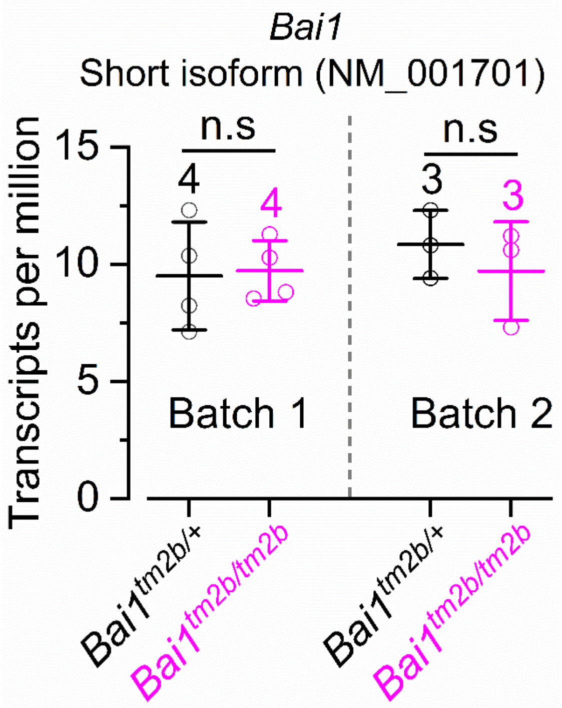

Supplementary Figure 2: The short isoform of Bai1 is not affected in the *Bai1* knockout mice. Related to Figurer 1.

Normalized counts for the short isoform of *Bai1* from batch 1 (4 pool samples for genotype) and batch 2 (3 pool samples for each genotype) of the RNA-sequencing experiments (see also **Figure 7**). Each sample contains both cochleae from 3-4 mice. Paired differential expression test was taken from DESeq2. Data are reported as mean ± SD. Statistical analysis was performed using the Tukey's post-test (one-way ANOVA). n.s. – not significant (Batch 1: P = 09981; Batch 2: P = 0.8731).

Supplementary Figure 3

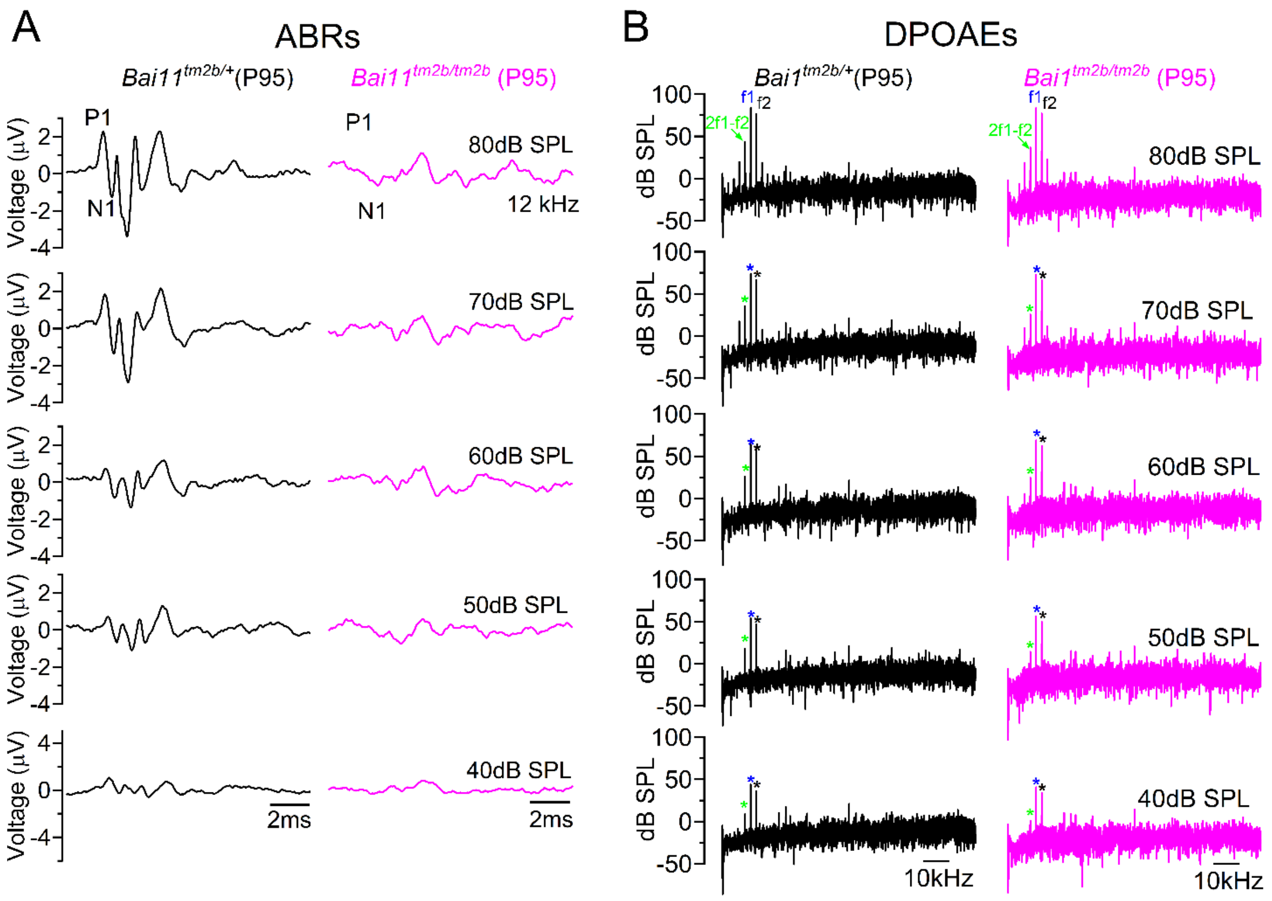

Supplementary Figure 3: ABRs and DPOAEs recorded from *Bai1* adult mice. Related to Figure 2.

A, ABR waveform responses at 12 kHz and increasing stimulus intensity (dB SPL) from P95 $Bai1^{tm2b/+}$and littermates $Bai1^{tm2b/tm2b}$ mice. Dashed lines indicate the position of wave I (P1 and N1: see Figure 2). **B**, DPOAE waveform responses at around 12 kHz and increasing stimulus intensity (dB SPL) from the same P95 $Bai1^{tm2b/+}$and $Bai1^{tm2b/tm2b}$ mice shown in panel **A**.

Supplementary Figure 4

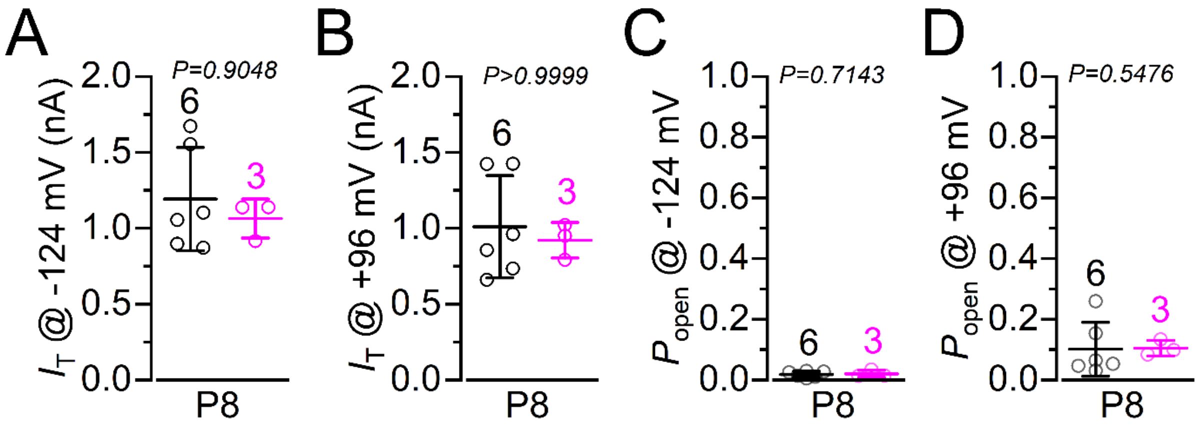

Supplementary Figure 4: Mechanoelectrical transduction in IHCs from *Bai1* mice. Related to Figure 3.

A,B, Maximal size of the MET current in the IHCs from both genotypes measured at –124 mV (**A**) and +96 mV (**B**) at P8 the age investigated in **Figure 3A,B**. **C,D**, Resting open probability (P_o) of the MET current in IHCs from the two genotypes at the holding of –124 mV (**C**) and +96 mV(**D**). The resting current is given by the holding current minus the current present during inhibitory bundle deflection. Control, 6 IHCs from 2 mice; *Bai1*$^{tm2b/tm2b}$, 3 IHCs from 1 mouse. All statistical comparisons shown above the panels **A-D** were carried out using a Mann-Whitney U test.

Supplementary Figure 5

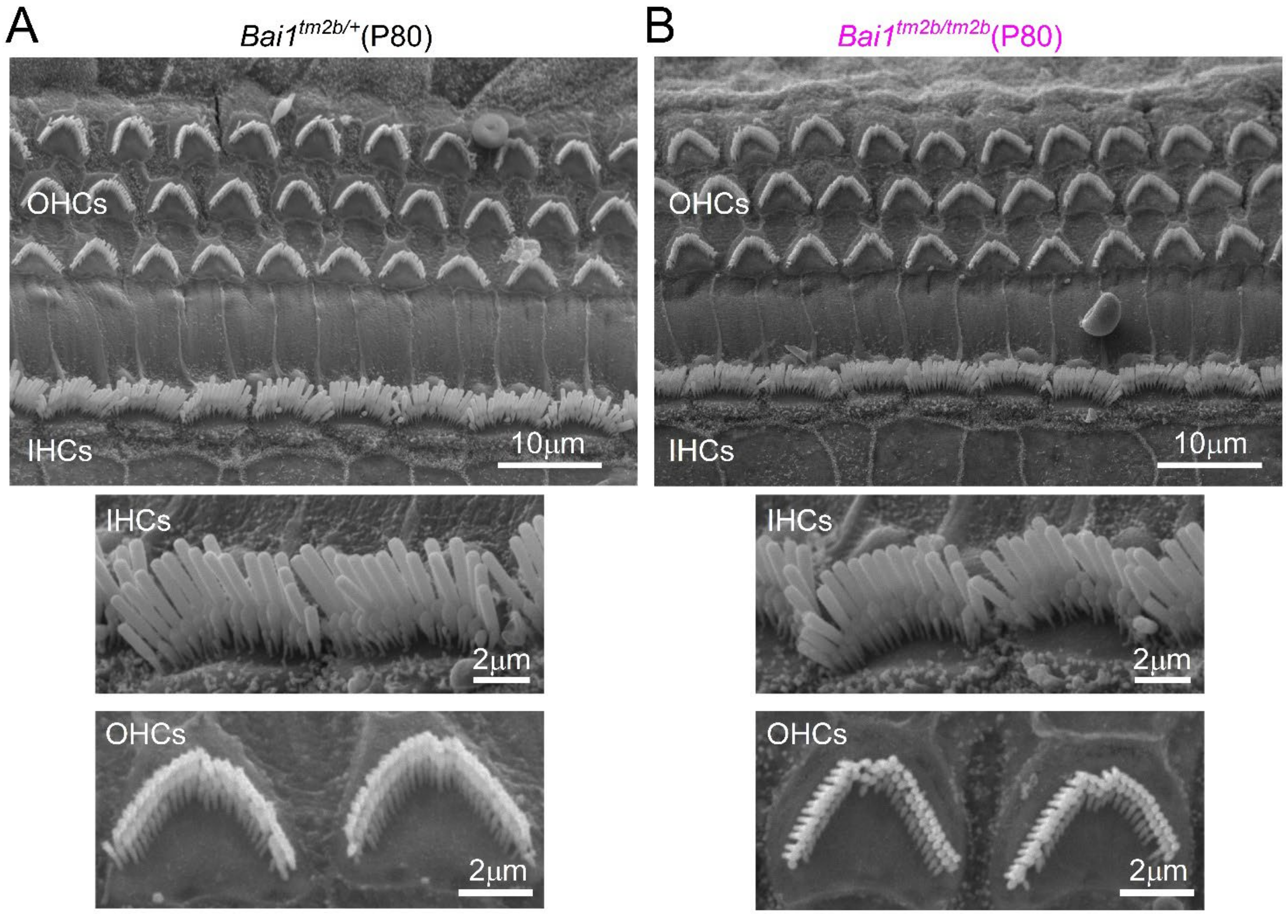

Supplementary Figure 5. The hair bundle structure of cochlear hair cells is normal in *Bai1* mice. Related to Figure 3.

A,B, Scanning electron micrographs (SEM) showing the typical hair bundle structure from apical-coil IHCs and OHCs in control *Bai1*$^{tm2b/+}$ (**A**) and *Bai1*$^{tm2b/tm2b}$ (**B**) P80 mice (examples from 3 mice per genotype). Lower panels show an expanded view of 2 IHC and OHC hair bundles for each genotype. Note that the hair bundles of IHCs and OHCs are composed of three rows of stereocilia in both genotypes.

Supplementary Figure 6

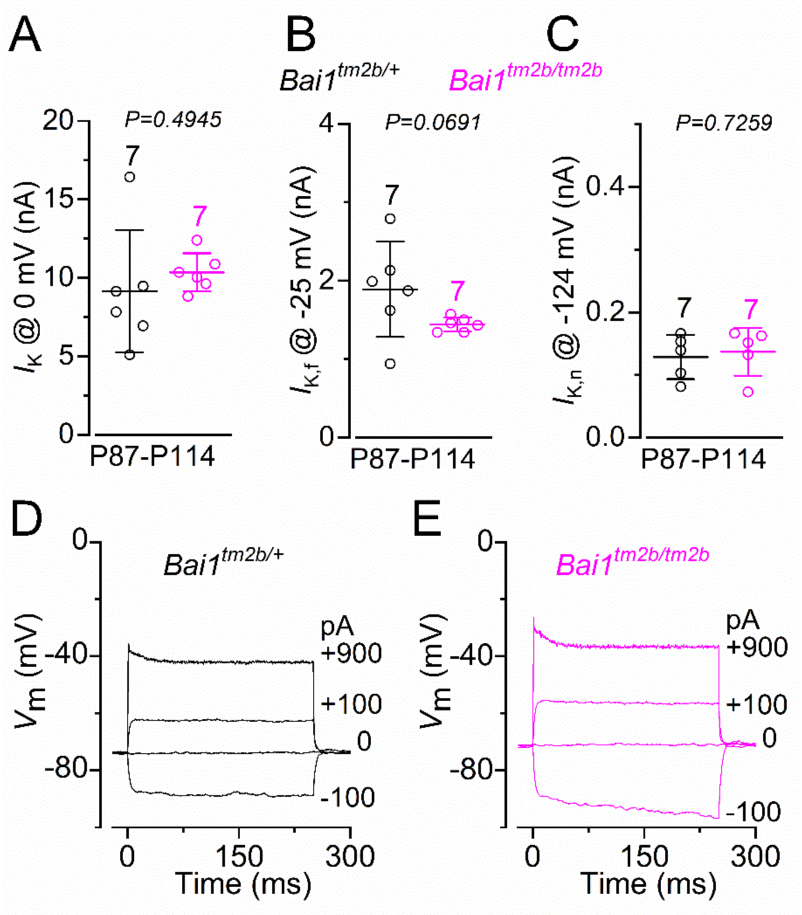

Supplementary Figure 6: Voltage and current clamp analysis from IHCs of adult *Bai1* mice. Related to Figure 3.

A, Size of the total steady-state outward K^+ current I_K recorded from adult IHCs (P87-P114) of both control (*Bai1*$^{tm2b/+}$) and homozygous (*Bai1*$^{tm2b/tm2b}$) mice. I_K was measured at the end of the voltage step from recordings as those shown in **Figure 3F,G**. **B**, Size of the large conductance Ca^{2+}-activated K^+ (BK) current $I_{K,f}$ recorded from IHCs of *Bai1*$^{tm2b/+}$and *Bai1*$^{tm2b/tm2b}$, which was measured at –25 mV and at 1 ms from the onset of the voltage step. ***C***, Size of the negatively activated K^+ current $I_{K,n}$ recorded from IHCs of mice from both genotypes. $I_{K,n}$ was measured as the difference between the peak and steady state of the deactivating inward current at -124 mV. In panels **A-C**, number of IHCs recorded from IHCs of P87-P114 mice are shown above each column and statistical comparisons were done using a Mann-Whitney U test. Data are shown as mean ± SD. **D,E**, Typical voltage responses from IHCs of *Bai1*$^{tm2b/+}$and *Bai1*$^{tm2b/tm2b}$. From these recordings we have measured the resting membrane potential of IHCs shown in **Figure 3I.** Control, 7 IHCs from 4 mice; *Bai1*$^{tm2b/tm2b}$, 7 IHCs from 3 mice.

Supplementary Figure 7

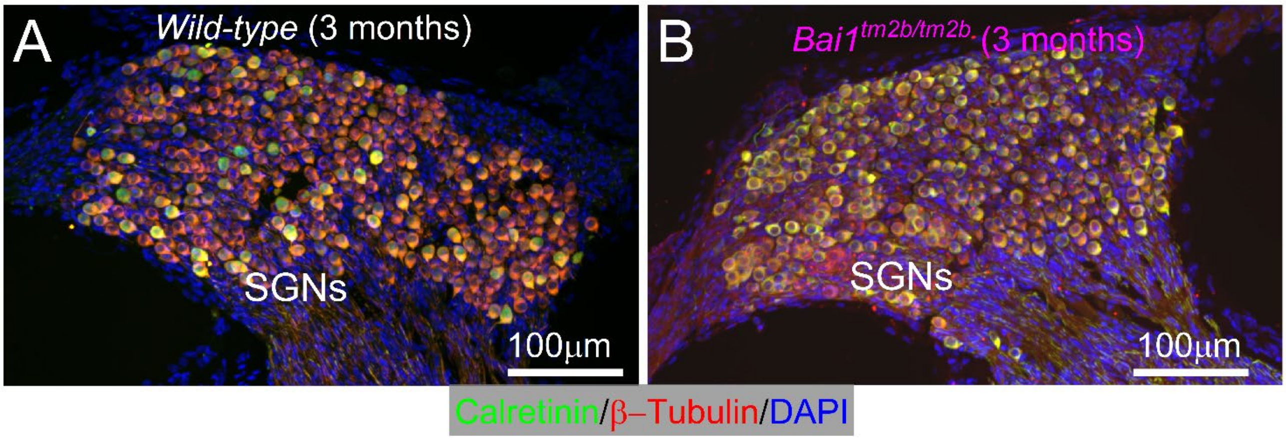

Supplementary Figure 7: Spiral ganglion neurons (SGNs) in *Bai1* mice. Related to Figure 3. **A,B**, Maximum intensity projections of confocal z-stacks of SGNs taken from cochlear cryosections of 3 month old control (**A**) and *Bai1*$^{tm2b/tm2b}$ (**B**) mice (examples from 3 mice per genotype). The cochlea was immunolabelled using antibodies against calretinin (SGN marker for most subtypes: green) and β-tubulin (afferent and efferent fibre marker: red). DAPI was used for nuclear staining. As expected from the presence of the afferent terminals onto the IHCs (**Figure 3N,O**), both genotypes retains SGNs.

Supplementary Figure 8

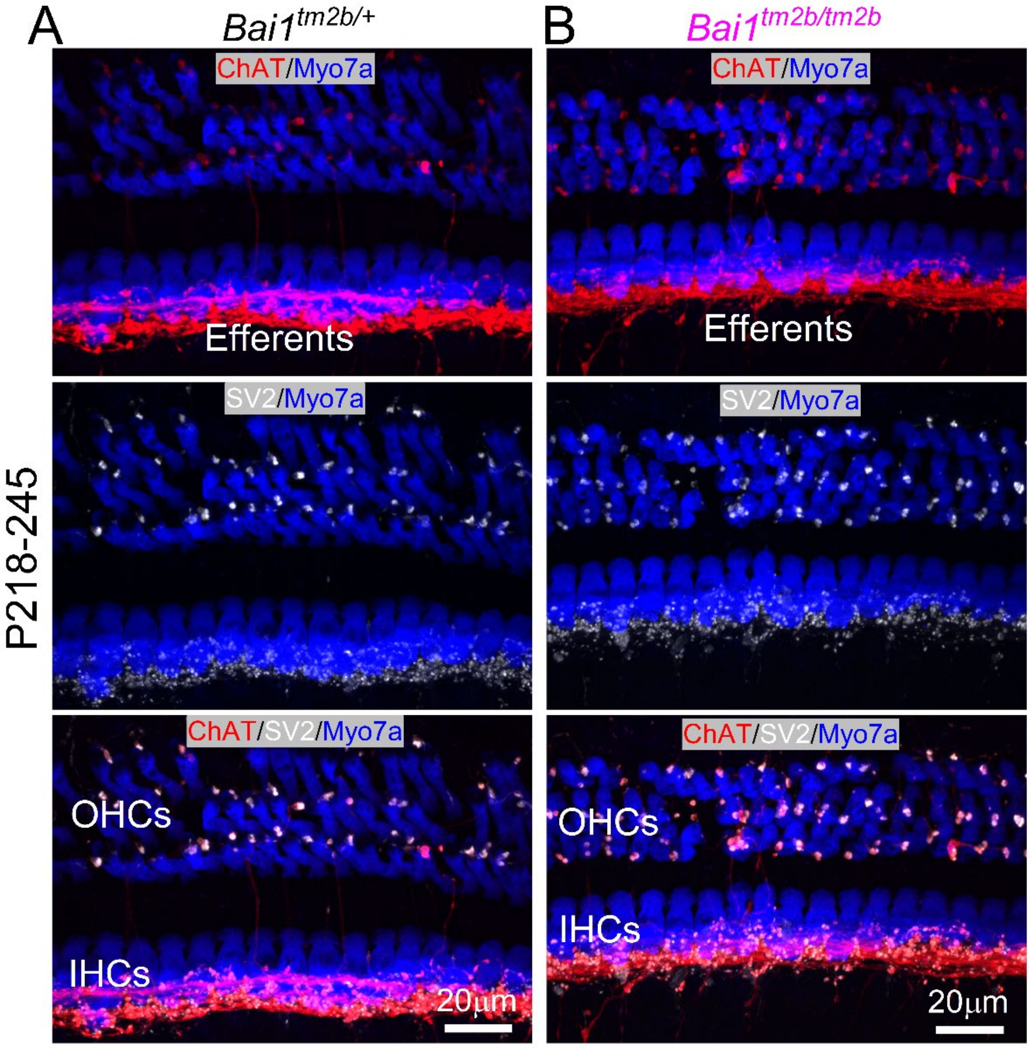

Supplementary Figure 8: Efferent fibres appear normal in adult *Bai1* mice. Related to Figure 3.

A, B, Maximum intensity projections of confocal z-stacks taken from the apical cochlear region of control (*Bai1*$^{tm2b/+}$) and homozygous (*Bai1*$^{tm2b/tm2b}$) mice at 7-8 months of age using antibodies against the synaptic vesicle protein 2 (SV2) present at the efferent endings (white) and efferent fibres ChAT (red). Myosin 7a (Myo7a) was used as a hair cell marker. Examples are from 3 mice per genotype. Due to the largely diffuse nature of the SV2 labelling at the IHC basal pole, we were unable to provide a reliable quantification.

Also note that the space between the IHCs and the OHCs is primarily defined by the supporting cells called "pillar cells", which do not express BAI1. The variable distance between the IHCs and OHCs between the two genotypes is due to the different orientation of the cochlear preparation, which may vary among experiments.

Supplementary Figure 9

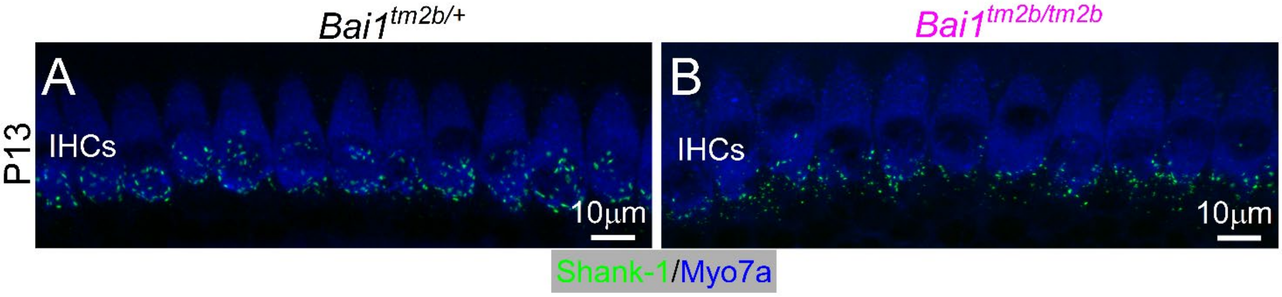

Supplementary Figure 9: Expression of the post-synaptic protein Shank-1 in *Bai1* mice. Related to Figure 4.

A,B, Maximum intensity projections of confocal z-stacks of IHCs taken from the apical cochlear region of control *Bai1*$^{tm2b/+}$ (**A**) and littermate *Bai1*$^{tm2b/tm2b}$ (**B**) mice using antibodies against the post-synaptic protein Shank-1. Myosin 7a (Myo7a) was used as the IHC marker. Examples are from 3 mice per genotype.

Supplementary Figure 10

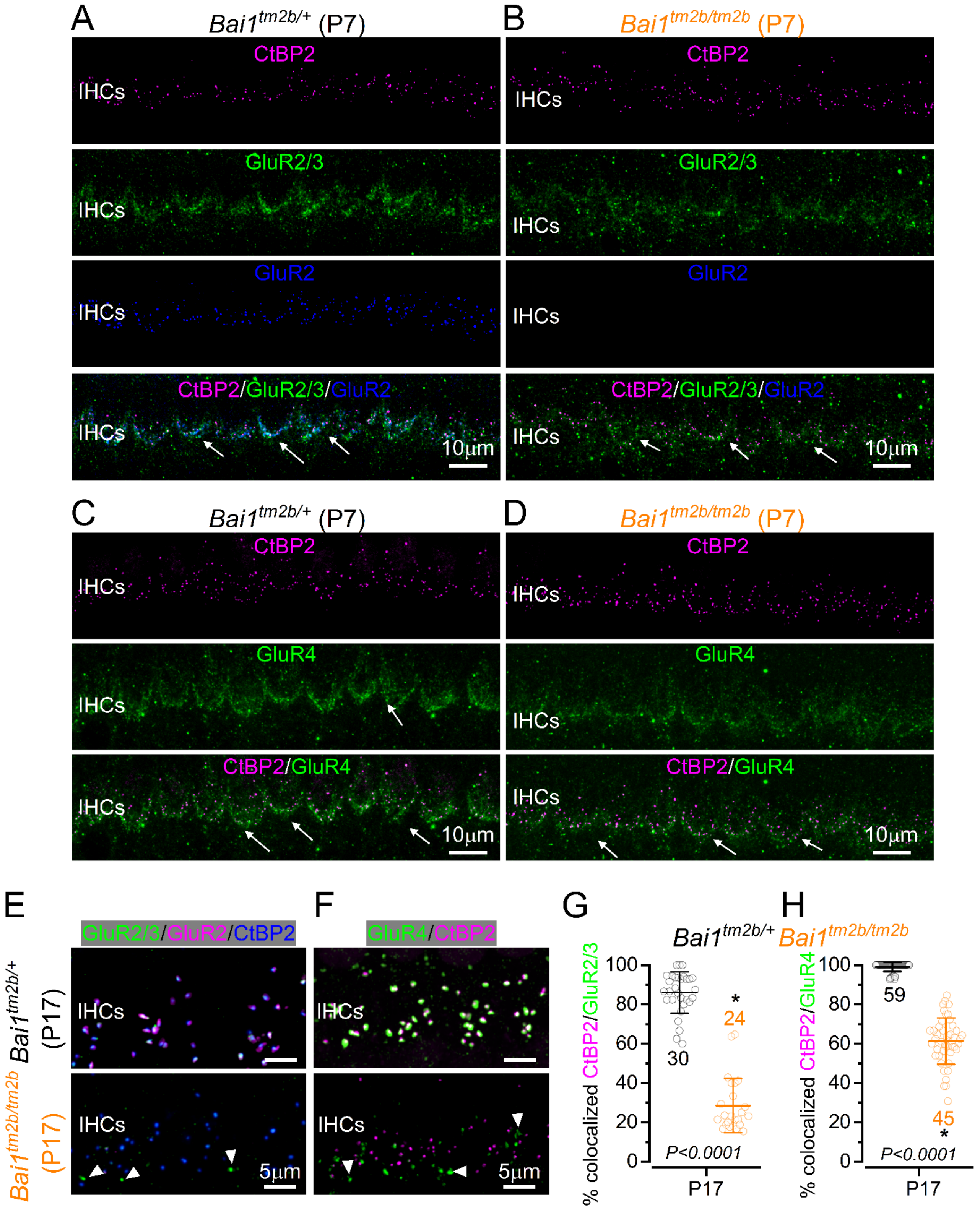

Supplementary Figure 10: Expression of AMPA-type GluR2/3 and GluR4 receptors in the IHCs of pre-hearing *Bai1* mice. Related to Figure 6.

A,B, Maximum intensity projections of confocal z-stacks of the synaptic region of the IHCs taken from the apical cochlear coil of control *Bai1*$^{tm2b/+}$ (**A**) and littermate *Bai1*$^{tm2b/tm2b}$ (**B**) P7 mice using antibodies against CtBP2 (ribbon synaptic marker) and both GluR2 and GluR2/3 (postsynaptic markers). The only available antibody against GluR3 also detects GluR2 (referred to as GluR2/3), the expression of GluR3 can only be identified in the IHCs from *Bai1*$^{tm2b/tm2b}$ since they do not express

GluR2. Arrows indicated the synaptic region of some of the IHCs from both genotypes. **C,D**, Images of the IHC synaptic region obtained as described in panels **A,B** from *Bai1*$^{tm2b/+}$ (**C**) and littermate *Bai1*$^{tm2b/tm2b}$ (**D**) P7 mice using antibodies against CtBP2 and GluR4 (postsynaptic markers). Arrows indicated the synaptic region of some of the IHCs from both genotypes.
Note that at P7 the distribution of GluRs is quite diffused around the IHC basal pole, making any puncta-like quantification unreliable. However, the puncta-like expression of GluRs in *Bai1*$^{tm2b/tm2b}$ mice seemed to be reduced or not well-clustered at the synaptic region in all samples investigated compared to control mice.

E,F, Maximum intensity projections of confocal z-stacks of the synaptic region of the IHCs taken from the apical cochlear coil of control *Bai1*$^{tm2b/+}$ (top panels) and littermate *Bai1*$^{tm2b/tm2b}$ (bottom panels) mice at P17. IHCs were labelled with antibodies against CtBP2 (ribbon synapse marker) alongside GluR2, GluR2/3 and GluR4 (postsynaptic markers). **G,H**, Percentage of colocalised CtBP2-GluR2/3 (**G**) and CtBP2-GluR4 (**H**) puncta at P17 from both genotypes. Data are plotted as mean values ± S.D and individual puncta count (smaller open symbols). Numbers above the mean data indicate the IHCs used for each genotype from 3 mice each (GluR2/3) and 4 and 3 mice (GluR4). Statistical values shown in panels **G,H** were obtained using a Mann-Whitney U test.

Supplementary Figure 11

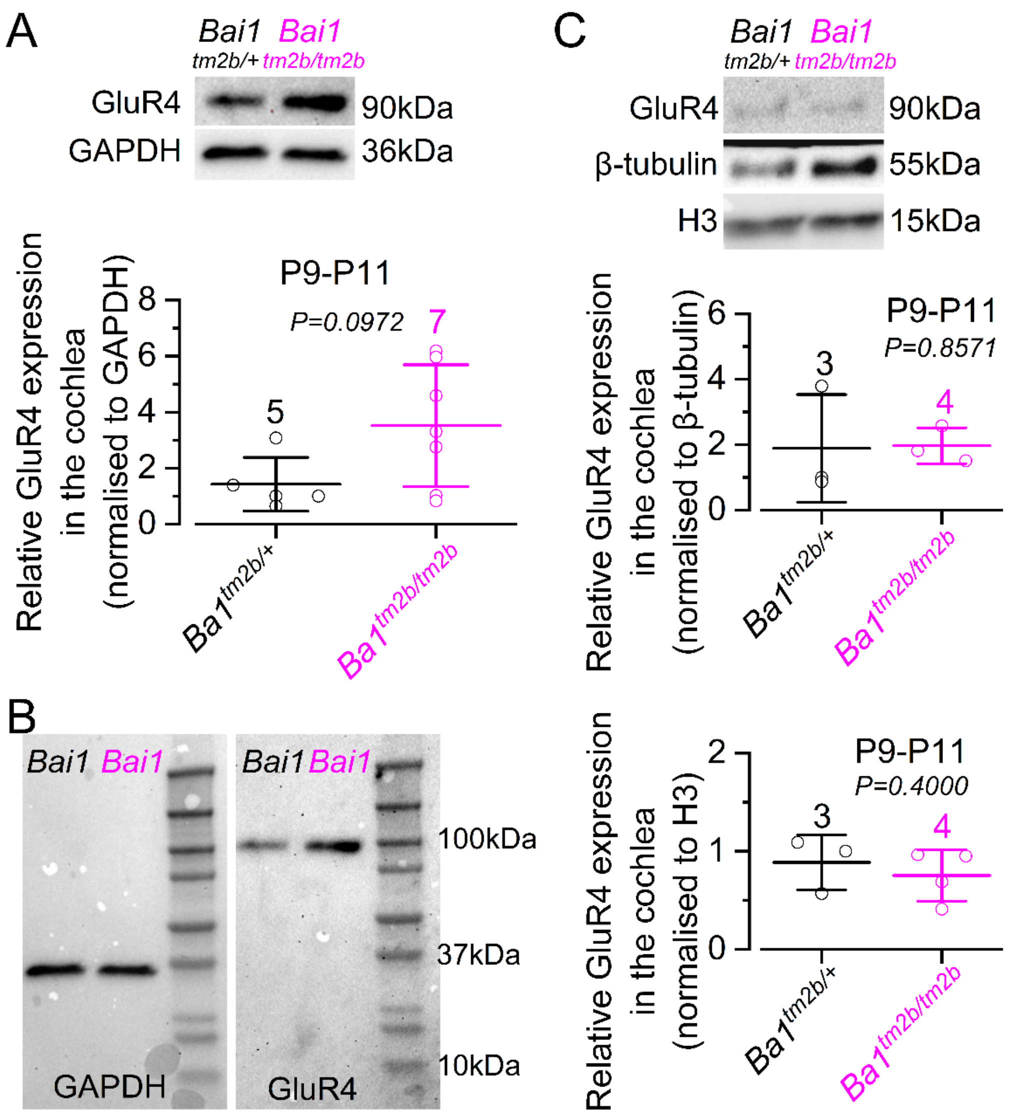

Supplementary Figure 11: GluR4 is present in the cochlea of *Bai1*-deficient mice. Related to Figure 6.

A, Western blot showing that GluR4 in present in mouse cochlea of control $Bai1^{tm2b/+}$ and $Bai1^{tm2b/tm2b}$ P9-P11 *Bai1*-deficient mice with GAPDH used as a loading control. **B,** Full gel run from one of the western blots performed showing both GluR4 and GAPDH. **C,** Expression of GluR4 normalised to additional loading controls: the neuronal marker β-tubulin and the nuclear marker Histone H3. Data was collected from 3 separate experiments from each genotype. Numbers above the data indicates biological replica (mice). Data are reported as mean ± SD. Statistical analysis was performed using Mann-Whitney U test.

Unfortunately, the very poor GluR2 signal in both control and $Bai1^{tm2b/tm2b}$ prevented a reliable quantification of this protein.

Supplementary Figure 12

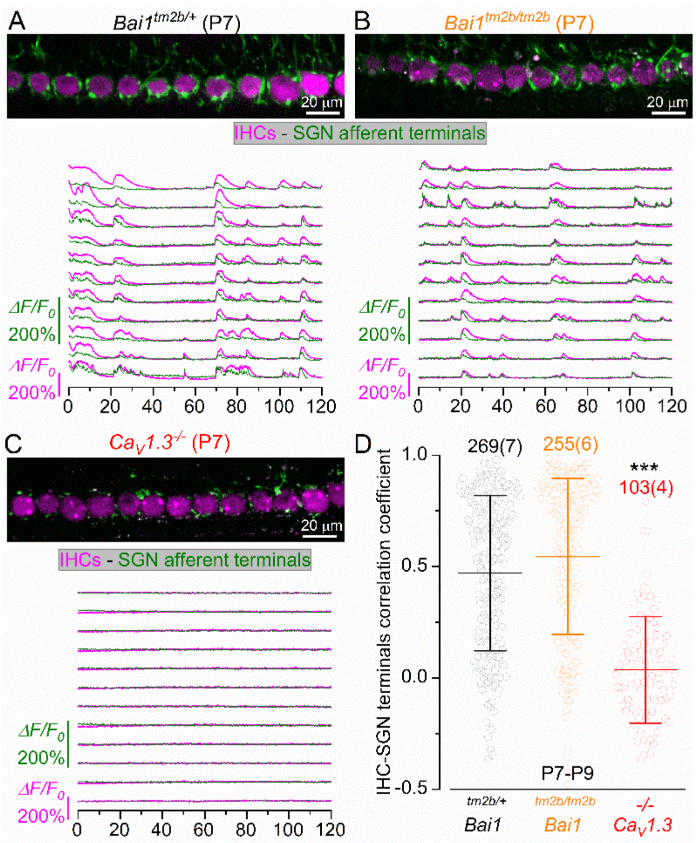

Supplementary Figure 12: Calcium transients in the afferent terminal of pre-hearing *Bai1*$^{tm2b/tm2b}$ mice. Related to Figures 5 and 6.

A-C, Representative $\Delta F/F_0$ traces from the IHCs (magenta) and SGN afferent terminals (green) from P7-P9 *Bai1*$^{tm2b/+}$ (**A**), *Bai1*$^{tm2b/tm2b}$ (**B**) and *Ca*$_V$*1.3*$^{-/-}$ (**C**) mice. IHC spontaneous Ca^{2+} transients elicited corresponding Ca^{2+} signals in the SGN afferent terminals in both *Bai1*$^{tm2b/+}$ and *Bai1*$^{tm2b/tm2b}$ mice. No Ca^{2+} signals were observed in both IHCs and postsynaptic terminals recorded from mice lacking the presynaptic Ca$_V$1.3 Ca^{2+} channels (**C**). Each set of traces (bottom to top) represent the recordings from all the IHCs and associated SGN terminals in the above image (left to right). All recordings were obtained at body temperature. Traces are computed as pixel averages of rectangular regions of interest centred on IHCs. IHCs were maintained in 0.3 mM Ca^{2+} to better mimic the predicted physiological endolymphatic Ca^{2+} concentration (Johnson et al., 2012). **D**, Average Pearson correlation coefficient between the Ca^{2+} activity in IHCs and in the SGN afferent terminals from the three mouse lines described above. As such, the SGN trace represent the average Ca^{2+} signal of all the afferent terminals contacting one IHC. No significantly different was found between *Bai1*$^{tm2b/+}$ and *Bai1*$^{tm2b/tm2b}$ (P = 0.0837; Kruskal Wallis, followed by Dunn test with Bonferroni correction), which were significantly higher than in *Ca*$_V$*1.3*$^{-/-}$ mice (P < 0.0001 with both *Bai1*$^{tm2b/+}$ and *Bai1*$^{tm2b/tm2b}$). Number of SGN terminals and (mice) is shown above the data.

Johnson SL, Kennedy HJ, Holley MC, Fettiplace R, Marcotti W (2012). The resting transducer current drives spontaneous activity in prehearing mammalian cochlear inner hair cells. J Neurosci 32:10479-10483.

Table S2

Pathways Down DEGs P22

Term	Adjusted P-value	Genes				
**BioPlanet 2019**						
Neuronal system	1.3307433178612273E-8	HCN4;ACHE;KCNH5;SYT2;KCNK10;SYT1;KCNC2;CHRNA4;GLS2;KCNJ14;GJD2;STX1B;PANX2;RIMS1;GNAL;KCNS3;KCNN1;KCNQ5;SLC17A7;CACNG2;KCNJ3				
Potassium channels	5.727147878644756E-4	HCN4;KCNH5;KCNK10;KCNC2;KCNS3;KCNJ14;KCNN1;KCNQ5;KCNJ3				
Glutamate neurotransmitter release cycle	0.004096723230982121	RIMS1;SYT1;GLS2;SLC17A7				
Transmission across chemical synapses	0.04714240119270449	RIMS1;ACHE;GNAL;SYT1;CHRNA4;GLS2;SLC17A7;CACNG2;KCNJ3				
Glucose metabolism	0.05487869635272343	PYGM;ENO1;PYGL;ENO2;PFKP				
Neurotransmitter release cycle	0.05487869635272343	RIMS1;SYT1;GLS2;SLC17A7				
**GO Cellular Component 2021**						
voltage-gated potassium channel complex (GO:0008076)	3.630313011667999E-4	HCN4;KCNC2;KCNIP3;KCNS3;KCNJ14;KCNN1;KCNQ5;KCNJ3				
potassium channel complex (GO:0034705)	0.003406955363701417	KCNC2;KCNIP3;KCNS3;KCNJ14;KCNN1;KCNQ5;KCNJ3				
exocytic vesicle membrane (GO:0099501)	0.004294304388582688	SV2C;SYT2;SYT1;SLC17A6;SLC17A7				
synaptic vesicle membrane (GO:0030672)	0.004294304388582688	SV2C;SYT2;SYT1;SLC17A6;SLC17A7				
neuron projection (GO:0043005)	0.011740669593695461	RTN4R;SYT2;SYT1;KCNC2;CHRNA4;MYO5A;ANK2;CACNA1H;CALB2;SV2C;SCN8A;ADGRB1;SACS;TMEM108;KCNN1;RGS8;NEFH				
voltage-gated sodium channel complex (GO:0001518)	0.023159965107777493	SCN8A;SCN4B;CACNA1H				
**Elsevier Pathway Collection**						
Dopamine Mediated Glutamate Release/Uptake Circle in Neuron in Migraine	0.003955269362079344	0	0	37.03703703703704	415.2918215695668	RIMS1;SYT1;SLC17A6;SLC17A7
Dopamine Mediated Glutamate Release and Glutamate Uptake Circle	0.00836014314494736	0	0	20.825949367088608	195.052510656963	RIMS1;SYT1;SLC17A6;SLC17A7
Exocytosis: Vesicle Priming	0.00836014314494736	0	0	20.825949367088608	195.052510656963	RIMS1;SYT1;CADPS;CACNG2

Pathways Up DEGs P22

Term	Adjusted P-value	Genes
**BioPlanet 2019**		
**GO Cellular Component 2021**		
No significant terms		
**Elsevier Pathway Collection**		
Proteins Involved in Hearing Loss	0.06604044517909133	TECTB;CLDN11;SALL1;CLDN14;OTOF;CHRNA10;ESPN;FGFR3
